# Uncoupling interferons and the interferon signature explains clinical and transcriptional subsets in SLE

**DOI:** 10.1016/j.xcrm.2024.101569

**Published:** 2024-05-13

**Authors:** Eduardo Gómez-Bañuelos, Daniel W. Goldman, Victoria Andrade, Erika Darrah, Michelle Petri, Felipe Andrade

**Affiliations:** 1 Division of Rheumatology, The Johns Hopkins School of Medicine, Baltimore, MD 21224

**Keywords:** systemic lupus erythematosus, interferon, interferon signature

## Abstract

Systemic lupus erythematosus (SLE) displays a hallmark interferon (IFN) signature. Yet, clinical trials targeting type I IFN (IFN-I) have shown variable efficacy, and blocking IFN-II failed to treat SLE. Here, we show that IFN type levels in SLE vary significantly across clinical and transcriptional endotypes. Whereas skin involvement correlated with IFN-I alone, systemic features like nephritis associated with co-elevation of IFN-I, IFN-II, and IFN-III, indicating additive IFN effects in severe SLE. Notably, while high IFN-II/-III levels without IFN-I had a limited effect on disease activity, IFN-II was linked to IFN-I-independent transcriptional profiles (e.g., OXPHOS and CD8^+^*GZMH*^+^ cells), and IFN-III enhanced IFN-induced gene expression when co-elevated with IFN-I. Moreover, dysregulated IFNs do not explain the IFN signature in 64% of patients or clinical manifestations including cytopenia, serositis, and anti-phospholipid syndrome, implying IFN-independent endotypes in SLE. This study sheds light on mechanisms underlying SLE heterogeneity and the variable response to IFN-targeted therapies in clinical trials.

## Highlights

• IFN-I, IFN-II, and IFN-III fluctuate significantly over time in SLE
• The IFN signature does not predict elevated IFN activity levels in SLE
• IFN types alone and in combination define distinct clinical and molecular subsets in SLE
• IFN-dependent and -independent pathways define unique mechanistic endotypes in SLE

## Graphical abstract

**Figure:**
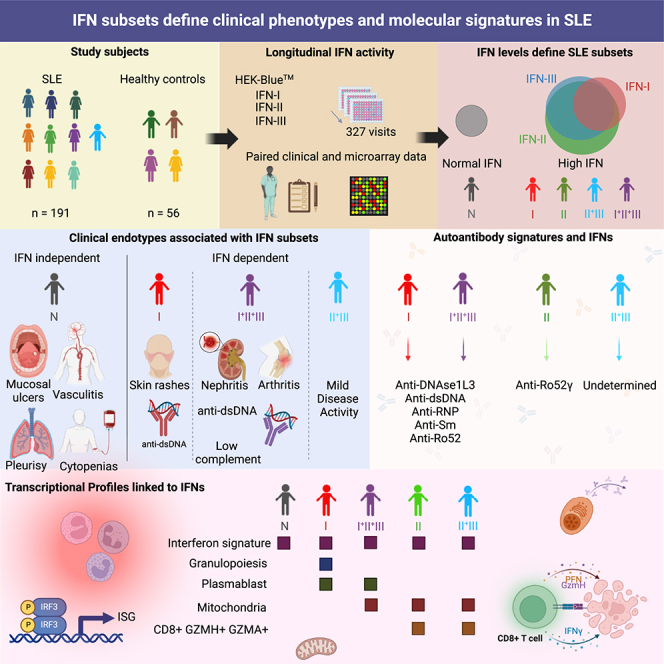

## Teaser

Gómez-Bañuelos et al. reveal that unique and interacting effects between IFN families, as well as IFN-independent mechanisms, contribute to distinct clinical endotypes, disease activity, and transcriptional signatures in SLE, thereby explaining disease heterogeneity and variable responses to IFN-targeted therapies in this complex disease.

## Introduction

Systemic lupus erythematosus (SLE) is a complex autoimmune disease affecting multiple organs that is characterized by marked clinical heterogeneity, a fluctuating course with relapses and remissions, and high-titer antibodies to diverse autoantigens.^1^ While the etiology of SLE remains unknown, bulk and single-cell transcriptomic profiling of peripheral blood and target tissues from patients with SLE have identified unique transcriptional signatures associated with the dysregulation of immune-related pathways, including a prominent transcriptional profile linked to increased signaling by interferons (IFNs).^2^^,^^3^^,^^4^^,^^5^^,^^6^^,^^7^^,^^8^^,^^9^^,^^10^

The IFN family comprises type I IFNs (IFN-Is; including 12 IFN-α subtypes plus IFN-β, IFN-ϵ, IFN-κ, and IFN-ω), the type II IFN (IFN-II; IFN-γ), and type III IFNs (IFN-IIIs; IFN-λ1–4).^11^ Although there is significant overlap in the induction of IFN-stimulated genes (ISGs) across members of all IFN families,^12^^,^^13^^,^^14^ genetic associations and biomarker studies have drawn attention to IFN-I as the primary pathogenic component in SLE.^15^ Clinical trials targeting the IFN-I pathway (either IFN-I-producing cells, soluble IFN-I, or the IFN-I receptor) in patients with SLE, however, have shown efficacy only in a subset of patients expressing the IFN signature.^16^^,^^17^^,^^18^^,^^19^^,^^20^^,^^21^ While disease heterogeneity is thought to be the main determinant of the variable response to IFN-I inhibition in SLE,^22^ the data also imply that the IFN signature may not be the optimal biomarker for identifying patients who will benefit from anti-IFN-I therapy. Indeed, significant gaps remain about the origin of the IFN signature in SLE and its relationship with IFN families other than IFN-I. Although several studies have shown that members of all IFN families are elevated in SLE,^23^^,^^24^^,^^25^^,^^26^^,^^27^ implying a role of the IFN-II and IFN-III in SLE pathogenesis, these studies lack parallel analysis of IFN levels with transcriptional profiling and clinical manifestations. Therefore, the unique and interactive contributions of the different IFN families to the IFN signature, clinical endotypes, and disease activity in SLE remain unclear.

An additional problem in the study of IFNs in SLE has been defining the best method for quantification. Except for IFN-II, which only includes IFN-γ, IFN-I and IFN-III comprise multiple subtypes, making it difficult to quantify them individually or in bulk with a single assay. While new technologies have been used to overcome this limitation and measure the majority of IFN-αs in SLE using a single assay (e.g., single-molecule array [Simoa] digital ELISA technology),^27^^,^^28^ other IFN-I members that may have functional relevance in SLE were excluded (e.g., IFN-β, IFN-ϵ, IFN-κ, and IFN-ω). More importantly, not all IFN-α subtypes have the same activating effect on cells. According to their potency, IFN-αs can be classified as having low (IFN-α1), intermediate (IFN-α2a, -4a, -4b, -5, -16, and -21), and high (IFN-α2b, -6, -7, -8, -10, and -14) activity.^29^ Since IFN-α subset levels are likely to vary among patients with SLE and over time, it is expected that similar circulating amounts of total IFN-α will have variable activities according to the proportion of IFNs with low, intermediate, and high activity. This notion explains why bulk IFN-α levels and activity do not correlate in a significant number of healthy controls and patients with SLE.^27^^,^^30^

Regarding other IFNs, the relationship between IFN-III levels and SLE is one of the most inconsistent, including divergences in the association with disease activity, clinical features, and the subset of elevated IFN-III (i.e., λ1, λ2, or λ3).^26^^,^^31^^,^^32^^,^^33^^,^^34^^,^^35^ These discrepancies are most likely due to differences in the methods used to quantify IFN-III and, importantly, the subtype of IFN selected as “exemplary” to reflect levels of all four IFN-IIIs.

An alternative approach to studying the effect of IFNs in SLE has been the use of functional assays, where reporter cells are exposed to serum/plasma and the IFN activity is determined by the transcriptional expression of ISGs or a reduction in the cytopathic effect of viruses.^23^^,^^27^^,^^36^ Since members of the same IFN family can only signal through a single receptor,^37^ this method is convenient because it reports on the coordinated activity of members of a single IFN family. Therefore, functional assays are more precise than quantifying bulk or individual amounts of IFN subtypes in capturing the effector function of circulating IFNs at the cellular level in SLE. However, current functional IFN assays are laborious, have only been applied for the study of IFN-I,^23^^,^^36^^,^^38^ and lack specificity because the reporter cells express receptors for the three IFN types.^39^^,^^40^

Here, to investigate the relationship between IFN families—individually and collectively—with clinical and transcriptional profiles in SLE, we used a unique and convenient cell-based reporter assay to quantify the specific activity of IFN-I, the IFN-II, and IFN-III in combination with clinical and whole-blood transcriptional data from a large prospective cohort of patients with SLE. Overall, the data indicate that the unique and interacting effects of the different IFN families, as well as IFN-independent mechanisms, have distinct contributions to clinical endotypes, disease activity, and transcriptional pathways dysregulated in SLE, thereby explaining disease heterogeneity in this complex disease.

## Results

### Activity levels of IFN-I, IFN-II, and IFN-III are elevated in SLE

**Figure 1:**
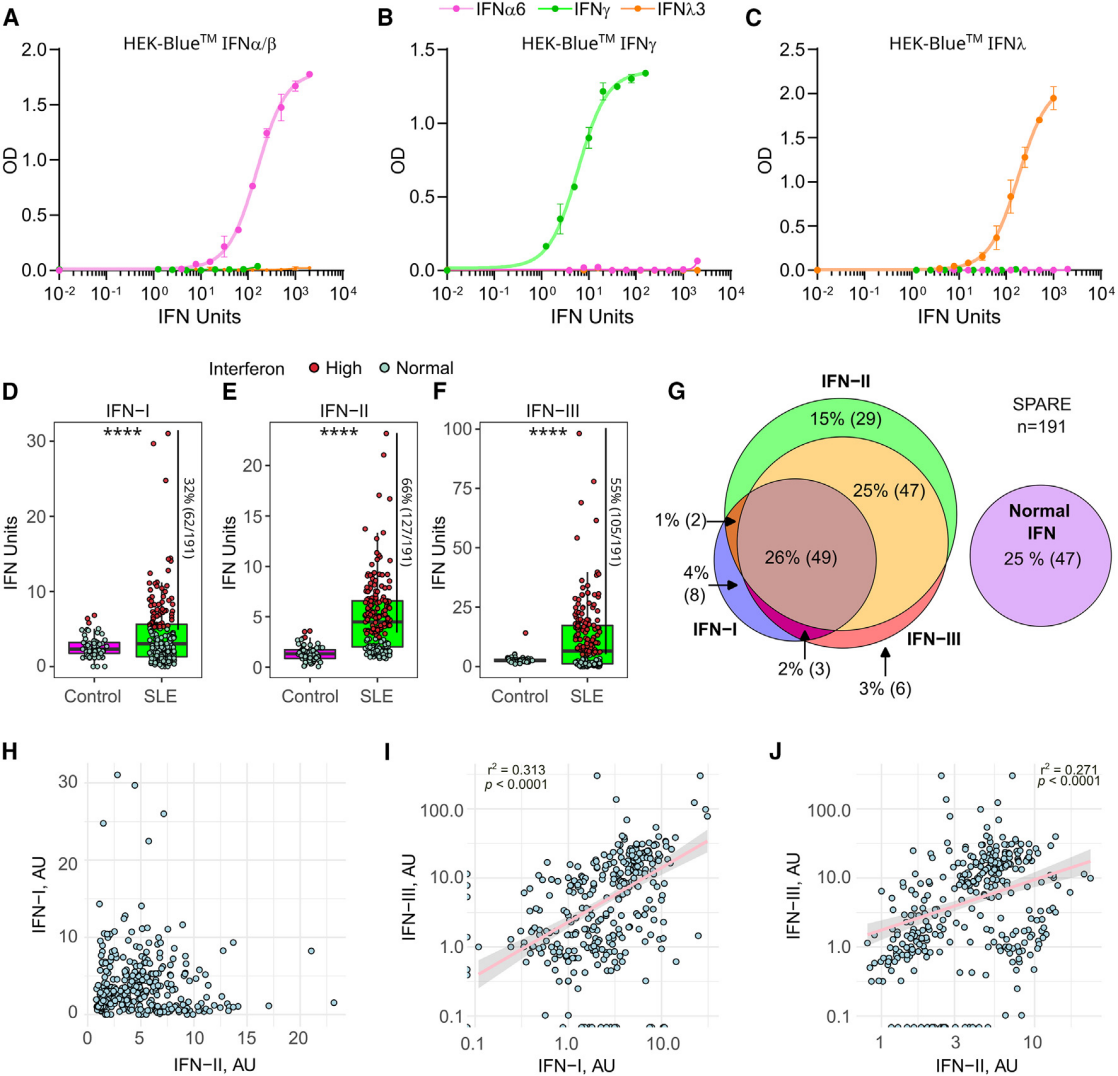
Activity levels of IFN-I, IFN-II, and IFN-III in patients from the SPARE cohort (A‒C) HEK-Blue IFN-α/β (A), HEK-Blue IFN-γ (B), and HEK-Blue IFN-λ (C) cells were incubated with increasing amounts of IFN-α6, IFN-γ, and IFN-λ3. The cells only detected their specific IFN with a range of 1.0–2,000 IU/mL for IFN-I and IFN-III and from 1 to 160 IU/mL for IFN-II. Experiments were performed in duplicate. Error bars represent the lower and upper values. (D‒F) Activity levels of IFN-I, IFN-II, and IFN-III in SLE (*n* = 191), and healthy controls (HCs) (*n* = 56). Whiskers indicate the lower and upper IFN values up to 1.5 times the interquartile range of the box. Samples with elevated levels of IFN-I, IFN-II, or IFN-III are colored in red. Cutoff was determined by ROC curve analysis with a 95% specificity vs. HCs. Elevated levels of IFN were set at 5.17, 2.9, and 5.0 IU/mL for IFN-I, IFN-II, and IFN-III, respectively. In patients with serial samples, only the first sample was included in the analysis. *p* values were obtained using Student’s t test. ∗∗∗∗*p* < 0.0001. (G) Venn diagram showing the intersection between elevated IFN types in patients with SLE. (H‒J) Correlation between increased activity levels of IFN-I, IFN-II, and IFN-III. The relationship between the IFN types was evaluated using linear regression.

Activity levels of human IFN types were quantified using commercial reporter cells engineered to express specific receptors and transcription factors to precisely detect bioactive human IFN-I, IFN-II, or IFN-III (InvivoGen). Similar to experimental data provided by the manufacturer, we validated that each cell line is specific for detecting its corresponding type of IFN (Figures 1A–1C). Moreover, using matched SLE serum and plasma, IFN activity levels showed a significant correlation (IFN-I, r^2^ = 0.866, *p* < 0.0001; IFN-II, r^2^ = 0.777, *p* < 0.0001; IFN-III, r^2^ = 0.745, *p* < 0.000.1) (Figure S1), indicating that blood processing has minimal effect on IFN concentrations, which is consistent with previous reports.^27^

Circulating activity levels of IFN types were then determined in 56 healthy controls and 191 patients with SLE from the “study of biological pathways, disease activity and response markers in patients with SLE” (SPARE).^41^^,^^42^^,^^43^ Demographic, clinical, and laboratory features of the SLE cohort are summarized in Table S1. IFN activity was detected both in healthy control and SLE samples (Figures 1D–1F). Compared to healthy controls, however, patients with SLE showed significantly higher activity of IFN-I, IFN-II, and IFN-III (mean units [SD], 2.6 [1.5] vs. 4.3 [4.5], 1.4 [0.8] vs. 5.0 [3.5], and 2.9 [1.7] vs. 11.3 (14.4), respectively, *p* < 0.0001 in all cases) (Figures 1D–1F). Using a ROC curve to determine cutoff points for each IFN, elevation in IFN-II was the most prominent in SPARE (66%, 127/191), followed by IFN-III (55%, 105/191) and IFN-I (32%, 62/191) (Figures 1D–1F). Moreover, 22% (43/191) of patients with SLE showed elevation of only one type of IFN, 53% (101/191) had more than one type of IFN elevated, and 25% (47/191) had IFN activities within the range of healthy controls (termed normal IFN) (Figure 1G). While we found no correlation between activity levels of IFN-I and IFN-II (Figure 1H), there was a mild correlation between IFN-III and IFN-I (r^2^ = 0.313, *p* < 0.0001) (Figure 1I), as well as IFN-III and IFN-II (r^2^ = 0.271, *p* < 0.0001) (Figure 1J).

### Activity levels of IFN types have different patterns according to disease activity and treatment

**Figure 2:**
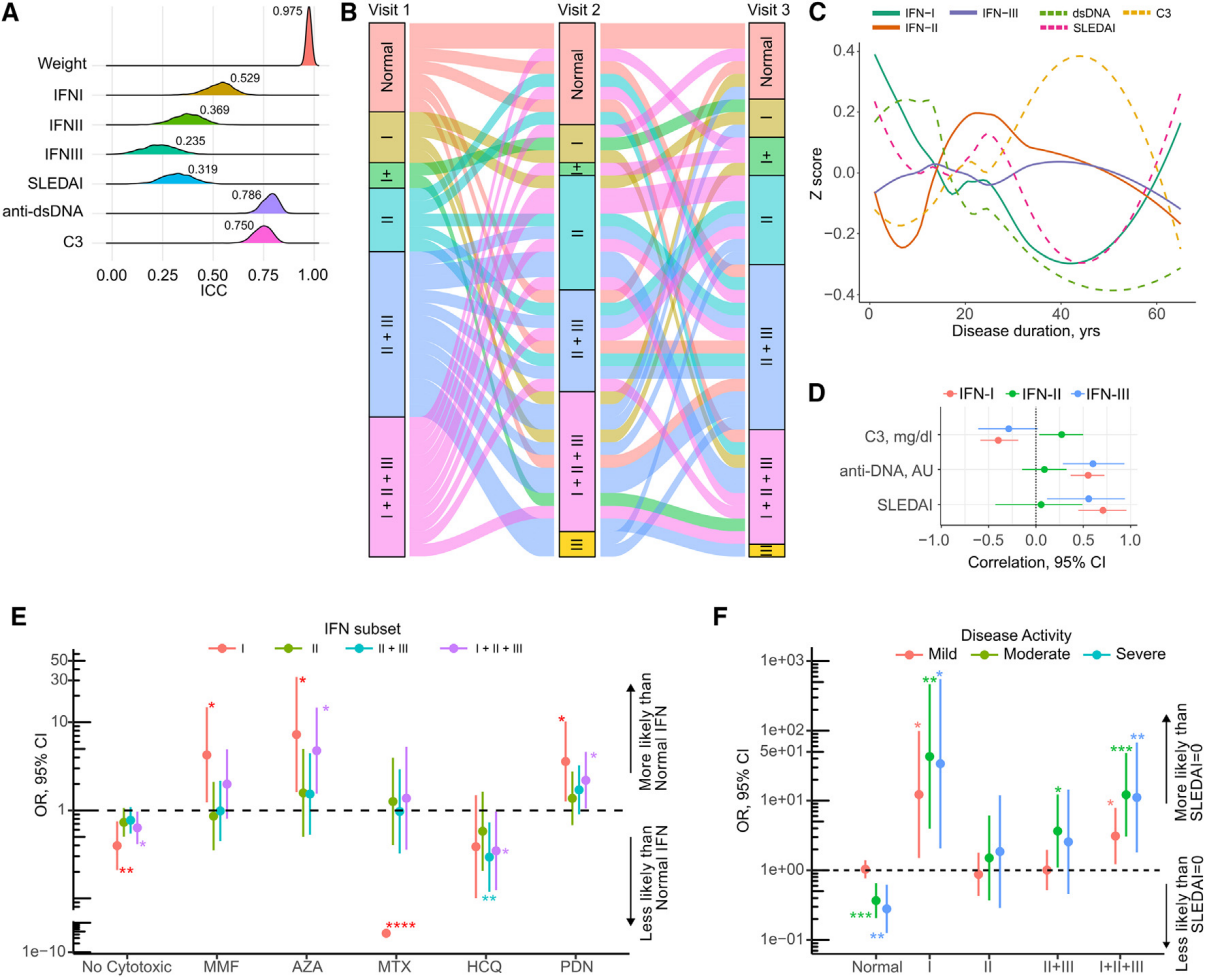
Longitudinal patterns of IFN type and their association with markers of disease activity and treatment in SLE (A) Intraclass correlation coefficient (ICC) of increased IFN activity levels in patients with SLE (*n* = 77) with more than 1 longitudinal sample. (B) Sankey diagram showing the flow of normal and increased IFN levels in patients with SLE (*n* = 42) with three longitudinal samples. Normal = normal IFN; I = high IFN-I; I+ = high IFN-I plus high IFN-II or IFN-III; II = high IFN-II; III = high IFN-III; II + III = high IFN-II + IFN-III; I + II + III = elevation of the three IFN types. Node height represents the proportion of patients with SLE with the indicated elevation of IFNs at the time of visit. Visit 1: normal, 17% (*n* = 7); I, 10% (*n* = 4); I+, 5% (*n* = 2); II, 12% (*n* = 5); II + III, 31% (*n* = 13); and I + II + III, 26% (*n* = 11). Visit 2: normal, 18% (*n* = 8); I, 7% (*n* = 3); I+, 2% (*n* = 1); II, 21% (*n* = 9); II + III, 19% (*n* = 8); I + II + III, 26% (*n* = 11); and III, 5% (*n* = 2). Visit 3: normal, 14% (*n* = 6); I, 7% (*n* = 3); I+, 3% (*n* = 7); II, 7% (*n* = 17); II + III, 31% (*n* = 13); I + II + III, 21% (*n* = 9); and III, 2% (*n* = 1). Each flow or link between the nodes represents a single patient. (C) Trajectory of elevated IFN types along with SLEDAI, C3, C4, and anti-dsDNA in patients with SLE. To represent grouped trajectories, *Z* scores of elevated IFN type and disease activity markers (SLEDAI, C3, and dsDNA) were projected over disease duration using loess-fitted curves. (D) Correlation between disease activity markers and elevated IFN type obtained by a Bayesian mixed-effects model in patients with SLE adjusted by disease duration. (E) Mixed-effects multinomial regression model examining the association between treatment and elevated IFN groups at the time of visit. (F) Mixed-effects multinomial regression model analyzing the relationship between normal and elevated IFN groups with SLE disease activity. Mild (SLEDAI = 1–2), moderate (SLEDAI = 2–9), severe (SLEDAI ≥10). In (C) and (D), the data are from 322 visits from 184 patients. In (E) and (F), the data are from the visits of 177 patients. Other elevated IFN groups (i.e., IFN-I + IFN-II, IFN-I + III, and IFN-III alone) were excluded in (E) and (F) due to the small number of patients in each group. MMF, mofetil mycophenolate; AZA, azathioprine; MTX, methotrexate; HCQ, hydroxychloroquine; PDN, prednisone.

To analyze the stability of the IFN types over time in SLE, we assessed the activity of the three IFN families in 226 longitudinal samples from 78 patients and calculated the intraclass correlation coefficient (ICC) to measure “within”-subject variability or repeatability using a linear mixed-effects model^44^ (Figure 2A). During follow-up, IFN-I exhibited the greatest repeatability among the three IFN types, followed by IFN-II and IFN-III (ICC [95% confidence interval (CI)], 0.529 [0.381, 0.648], 0.369 [0.224, 0.509], and 0.235 [0.085, 0.348]). Overall, all IFN types showed lower ICCs compared to body weight, which was the most stable parameter during follow-up (ICC [95% CI], 0.975 [0.962, 0.982] *p* < 0.0001), but were comparable to the SLE disease activity index (SLEDAI; ICC [95% CI], 0.319 [0.169, 0.464]) (Figure 2A), suggesting that, similar to disease activity, IFN levels are variable during the course of SLE.

To better depict the stability of IFNs over time, we selected a subset of patients with SLE (*n* = 42) who had IFN measurements in three consecutive visits (Figure 2B). Because most patients had elevated levels of more than one IFN type, they were divided into subsets based on the single or combined activity of the three IFN types. 90% (38/42) of patients with SLE changed their IFN subset through the three visits. Among these, 72% of patients with normal IFN levels at baseline developed a single or combined elevated IFN activity in the subsequent visits (Figure 2B), confirming that IFNs are highly dynamic over the course of the disease.

To determine whether the variability in IFN activity was associated with disease activity, we fitted a linear mixed-effects model using data from all patients with SLE. IFN-I and IFN-III were significantly associated with SLEDAI (exp(β) [95% CI], 1.06 [1.04, 1.08] and 1.06 [1.02, 1.1], respectively), IFN-II was associated with longer disease duration, and IFN-I was negatively associated with disease duration (exp(β) [95% CI], 1.01 [1.0, 1.01] and 0.99 [0.98, 0.99], respectively) (Tables S2–S4). To better understand the relationship between IFN activity levels and disease activity over time, we projected the IFN levels and disease activity parameters over disease duration (Figure 2C). Notably, in contrast to IFN-II and IFN-III, IFN-I levels paralleled changes in SLEDAI, anti-double-stranded DNA (anti-dsDNA), and complement component 3 (C3) (Figure 2C). Using a Bayesian mixed-effects model to examine the correlation of individual IFN types with disease activity measures (Figure 2D), we confirmed that changes in IFN-I were linked with changes in SLEDAI, anti-dsDNA, and C3. In contrast, changes in IFN-II were associated with increased levels of C3, and IFN-III was only associated with changes in SLEDAI and anti-dsDNA.

Interestingly, when the data were modeled to address whether levels of IFNs were associated with treatment and disease control, we found that commonly used immunosuppressors had distinct effects on IFNs. Notably, IFN-I (either alone or in combination with IFN-II + IFN-III) was the main IFN significantly affected by treatment and disease activity (Figures 2E and 2F). In particular, patients without cytotoxic treatment and those receiving methotrexate or hydroxychloroquine were less likely to have increased levels of IFN-I and/or IFN-I + IFN-II + IFN-III. In contrast, the use of mycophenolate, azathioprine, or prednisone was associated with an increased probability of having elevation of IFN-I and/or IFN-I + IFN-II + IFN-III. Overall, increased levels of IFN-II alone or in combination with IFN-III were similar regardless of treatment, with the exception of hydroxychloroquine, which was the only therapy associated with decreased activity of IFN-II + IFN-III (Figure 2E). Similarly, neither increased IFN-II nor IFN-II + IFN-III levels were linked to disease activity (either mild, moderate, or severe), except for IFN-II + IFN-III, which was associated with moderate activity (Figure 2F). In contrast, increased activity levels of single IFN-I and IFN-I + IFN-II + IFN-III were always present in patients with mild to severe disease activity. Patients with IFNs within a normal range were less likely to have active SLE (Figure 2F). Importantly, other elevated IFN groups (i.e., IFN-I + IFN-II, IFN-I + IFN-III, and IFN-III alone) were excluded from this and subsequent analyses due to the small number of patients in each group.

### IFN types define distinct clinical subsets in SLE

**Figure 3:**
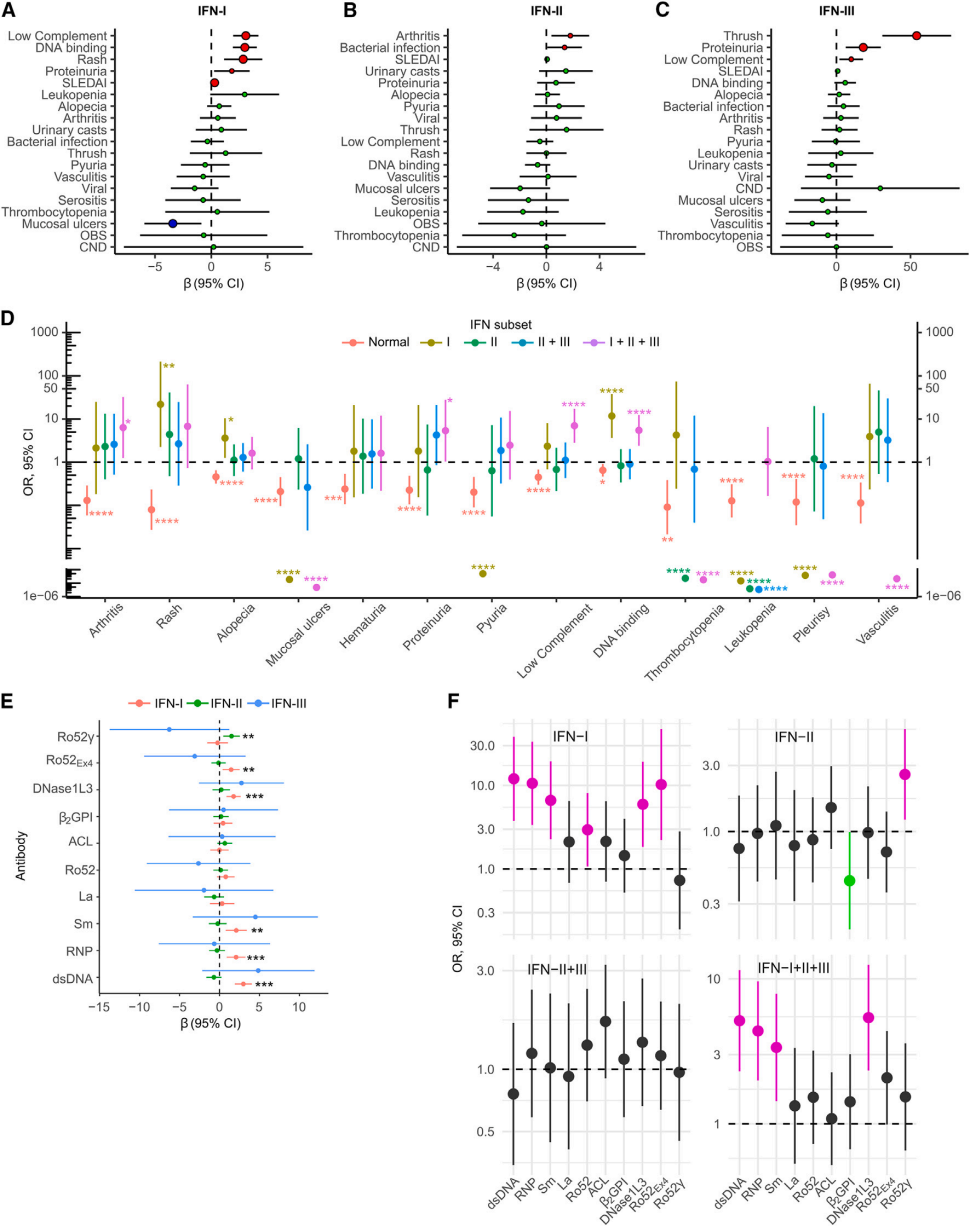
SLE disease subsets are predictive of increased activity levels of IFN types (A‒C) Effect of SLE activity features at time of visit on increased levels of IFN-I (A), IFN-II (B), and IFN-III (C). (D) Predictive value of normal and elevated IFN groups for specific SLEDAI items at the time of visit. (E) Increased activity levels of IFN-I, IFN-II, or IFN-III according to autoantibody positivity in SLE. (F) Predictive value of SLE autoantibodies for elevated IFN groups at the time of visit. In (D)–(F), other elevated IFN groups (i.e., IFN-I + IFN-II, IFN-I + IFN-III, and IFN-III alone) were excluded due to the small number of patients in each group. In (A)–(C) and (E), associations were evaluated using a mixed-effects linear regression model adjusted by disease duration. Data analysis included 322 visits from 184 patients. In (D) and (F), associations were evaluated using a mixed-effects multinomial logistic regression adjusted by disease duration. Data analysis included 300 visits from 177 patients. ∗*p* < 0.05, ∗∗*p* < 0.01, ∗∗∗*p* < 0.001, and ∗∗∗∗*p* < 0.0001.

To determine the clinical endotypes associated with IFNs, we analyzed the relationship between SLEDAI specific items and IFN activity levels (Table S5). Low complement, anti-DNA antibodies, rash, proteinuria, and SLEDAI were associated with higher IFN-I activity, while mucosal ulcers were associated with lower IFN-I activity (Figure 3A). Arthritis and recent bacterial infections were associated with increased IFN-II (Figure 3B), and oral candida, low complement, and renal involvement were associated with increased IFN-III activity (Figure 3C).

Given that most patients exhibit elevated levels of more than one IFN type (Figure 1G), a multinomial logistic regression model was used to evaluate the predictive value of individual and combined IFN groups across disease activity score items. The single elevation of IFN-I was associated with skin manifestations (rash and alopecia) and anti-DNA binding, while IFN-II elevation alone or in combination with IFN-III was not associated with any disease activity item (Figure 3D; Table S6). Interestingly, features of systemic disease—such as arthritis, anti-DNA antibodies, nephritis, and low complement—were only significantly associated with the co-elevation of the three IFN types (Figure 3D; Table S6), suggesting an additive effect of the three IFN families in severe SLE. In contrast, normal levels of IFNs were linked to a reduced frequency of any disease activity item (Figure 3D; Table S6). Remarkably, a subset of disease activity manifestations such as mucosal ulcers, thrombocytopenia, leukopenia, pleurisy, and vasculitis was not associated with increased activity of any IFN type. Rather, elevated levels of different IFNs were less likely to be associated with these manifestations (Figure 3D; Table S6).

Except for antibodies to Ro52, antibodies to dsDNA, ribonucleoprotein (RNP), Sm, and DNase1L3 were significantly associated with increased activity levels of IFN-I (Figure 3E). Interestingly, however, antibodies against Ro52Ex4 and Ro52γ—two recently described subsets of anti-Ro52 antibodies^45^—were linked to increased levels of IFN-I and IFN-II, respectively (Figure 3E). No autoantibody was associated with increased IFN-III activity (Figure 3E). Among the individual and combined groups of elevated IFNs, antibodies to dsDNA, RNP, Sm, and DNaseL3 were predictive of the single elevation of IFN-I, as well as the co-elevation of the three IFN types (Figure 3F). Antibodies to Ro52 and Ro52Ex4 were only significantly associated with increased activity levels of IFN-I alone, while antibodies to Ro52γ were the only autoantibodies linked to elevation of IFN-II alone (Figure 3F). Anti-phospholipid syndrome (APS) antibodies (i.e., anti-β2-glycoprotein I and anti-cardiolipin, anti-B2GPI, and anti-aCL, respectively) were not associated with any IFN type or IFN group. Rather, anti-B2GPI antibodies were protective of the single elevated IFN-II (Figure 3F).

### Transcriptional signatures associated with IFN types in SLE

**Figure 4:**
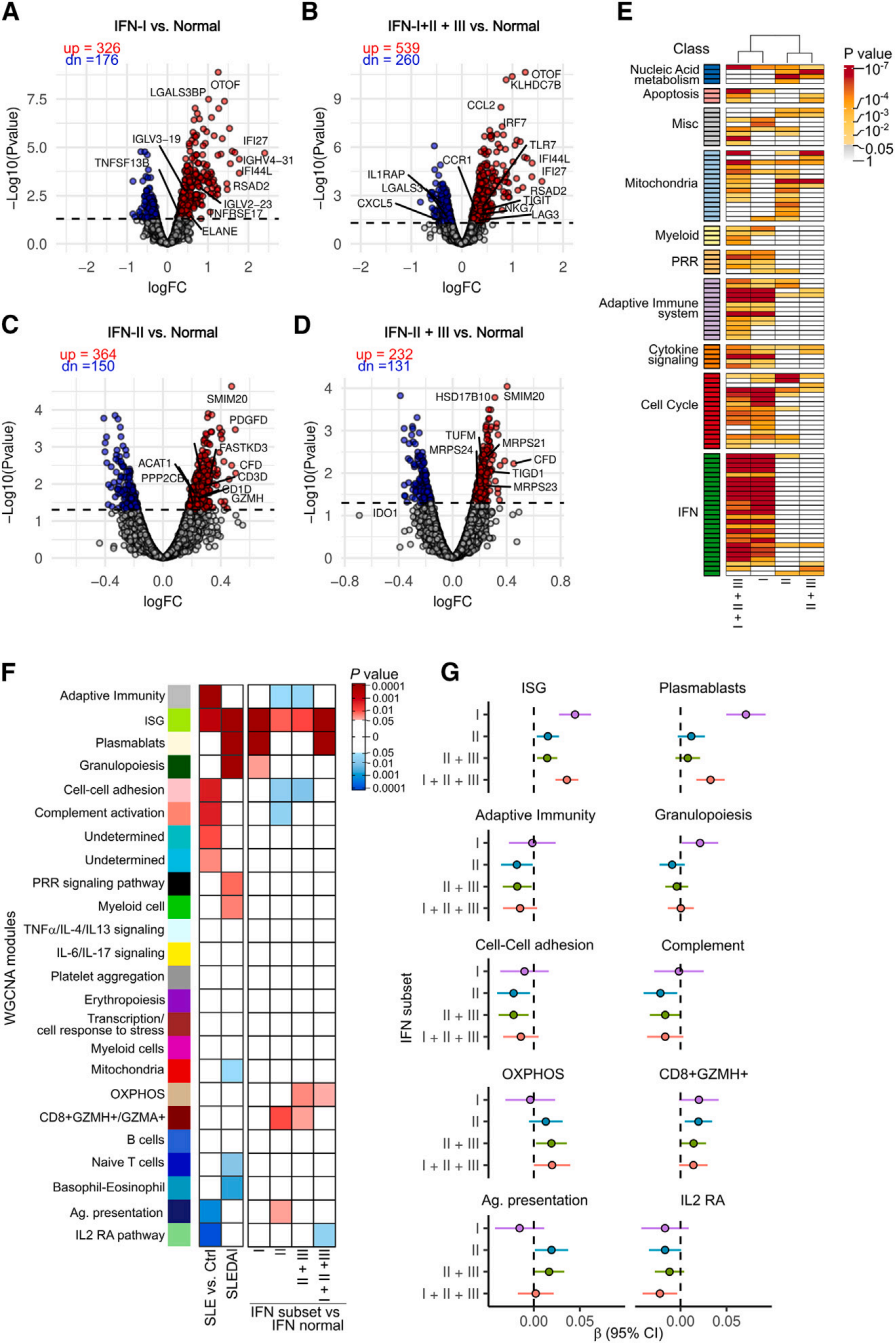
Increased activity levels of individual and combined IFN types are associated with distinct transcriptional profiles in SLE (A‒D) Differentially expressed transcripts (DETs) in SLE visits with single elevated IFN-I (A, *n* = 20), IFN-I + IFN-II + IFN-III (B, *n* = 48), single elevated IFN-II (C, *n* = 63), and IFN-II + IFN-III (D, *n* = 94) vs. normal IFN (*n* = 75). Differential expression analysis was done using a linear mixed-effects model to account for patients with repeated samples. DETs were determined when the adjusted *p* value was <0.05. The dotted line on each volcano plot equals an adjusted *p* value of 0.05. “up” and “dn” annotations correspond to the counts of upregulated (in red) or downregulated transcripts (in blue), respectively, from each comparison. Representative DETs from each subset are labeled. (E) Enriched pathways associated with increased activity levels of IFN groups. Enrichment was performed using the multi-gene-list meta-analysis feature from Metascape. (F) Association between gene co-expression modules with disease state (SLE vs. control), disease activity (SLEDAI), and increased activity levels of IFN groups. Shades of red or blue represent modules positively (β > 0) or negatively associated (β < 0) with the indicated variable. Associations were done using a linear mixed-effects model. (G) Effect size (β) of the IFN groups over significantly associated weighted correlation network analysis modules. In (F) and (G), the data are from 300 visits of 177 patients. In (A)–(G), other increased IFN groups (i.e., IFN-I + IFN-II, IFN-I + IFN-III, and IFN-III alone) were excluded due to the small number of patients in each group. PRR, pattern recognition receptor; pDC, plasmacytoid dendritic cell; ISG, IFN-stimulated genes; OXPHOS, oxidative phosphorylation.

Patients with SLE display unique blood transcriptional profiles, including a hallmark IFN signature.^2^ To determine whether distinct transcriptional fingerprints are associated with the activity of IFNs in SLE, we used gene expression data from blood samples collected in parallel with the samples used to measure IFN activities. Because clinical subsets in SLE are linked to individual and combined IFN type groups (Figure 3D), we initially performed differential gene expression analysis using a mixed-effects linear model between SLE subsets with elevated IFNs vs. those with normal IFN levels (Figures 4A–4D; Data S1). Then, to functionally interpret the transcriptomic differences across IFN type groups, we compared the enriched pathways between the differentially expressed transcripts (DETs) of each IFN group (Figure 4E; Data S1). Enrichment analysis revealed that increased activity levels of IFN-I alone were associated with pathways related to IFN activation, cytokine signaling, cell cycle, adaptive immune system, and pattern recognition receptors (PRRs) (Figure 4E; Data S1). In contrast, DETs associated with increased levels of IFN-II and IFN-II + IFN-III were enriched in transcriptional pathways linked to mitochondria and nucleic acid metabolism, and IFN-II + IFN-III was additionally associated with apoptosis (Figure 4E; and Data S1). Remarkably, the transcriptome associated with increased activity of IFN-I + IFN-II + IFN-III showed an overlap of pathways enriched in the IFN-I, IFN-II, and IFN-II + III subsets (Figure 4E; Data S1), which is consistent with immune activation by the combined effect of the three IFN families.

To gain further insights into the role of IFNs in transcriptional pathways dysregulated in SLE, we performed weighted correlation network analysis^46^ to define gene co-expression modules using gene expression data from 18 healthy controls and 327 samples from 191 patients with SLE. We identified 24 transcriptional modules (Data S2), which were classified according to their association with disease state (i.e., differentially regulated in SLE vs. controls) or disease activity (SLEDAI) (Figure 4F). Eight modules were associated with disease state with six upregulated (antigen presentation, ISGs, cell-cell adhesion, complement, undetermined turquoise, and undetermined cyan) and two downregulated (monocytes and interleukin-2 receptor α [IL-2-RA] pathway). Seven modules were significantly associated with disease activity, of which five were upregulated (ISGs, plasmablasts, granulopoiesis, PRRs, and myeloid cells) and three downregulated (mitochondria, naive T cells, and basophil-eosinophil) (Figure 4F).

To identify specific modules associated with increased levels of IFN type groups in SLE, we compared the activity of individual modules between the elevated IFN groups and normal IFN (Figures 4F and 4G). Increased levels of IFN-I alone and/or IFN-I + IFN-II + IFN-III were linked to the upregulation of disease activity modules (i.e., plasmablasts and granulopoiesis), as well as the prominent activation of ISGs (Figures 4F and 4G). In addition, the elevation of IFN-I + IFN-II + IFN-III was associated with increased activity of the oxidation phosphorylation module and downregulation of the IL-2-RA pathway. Interestingly, except for a mild association with ISG upregulation, increased activity of IFN-II alone and/or IFN-II + IFN-III showed a negative association with disease state modules (i.e., antigen presentation, cell-cell adhesion, and complement) and no association with any disease activity module. Instead, these IFN groups were linked to the upregulation of modules associated with oxidative phosphorylation and/or CD8^+^*GZMH*^+^ T cells (Figures 4F and 4G).

### Association between the IFN types and the IFN signature in SLE

**Figure 5:**
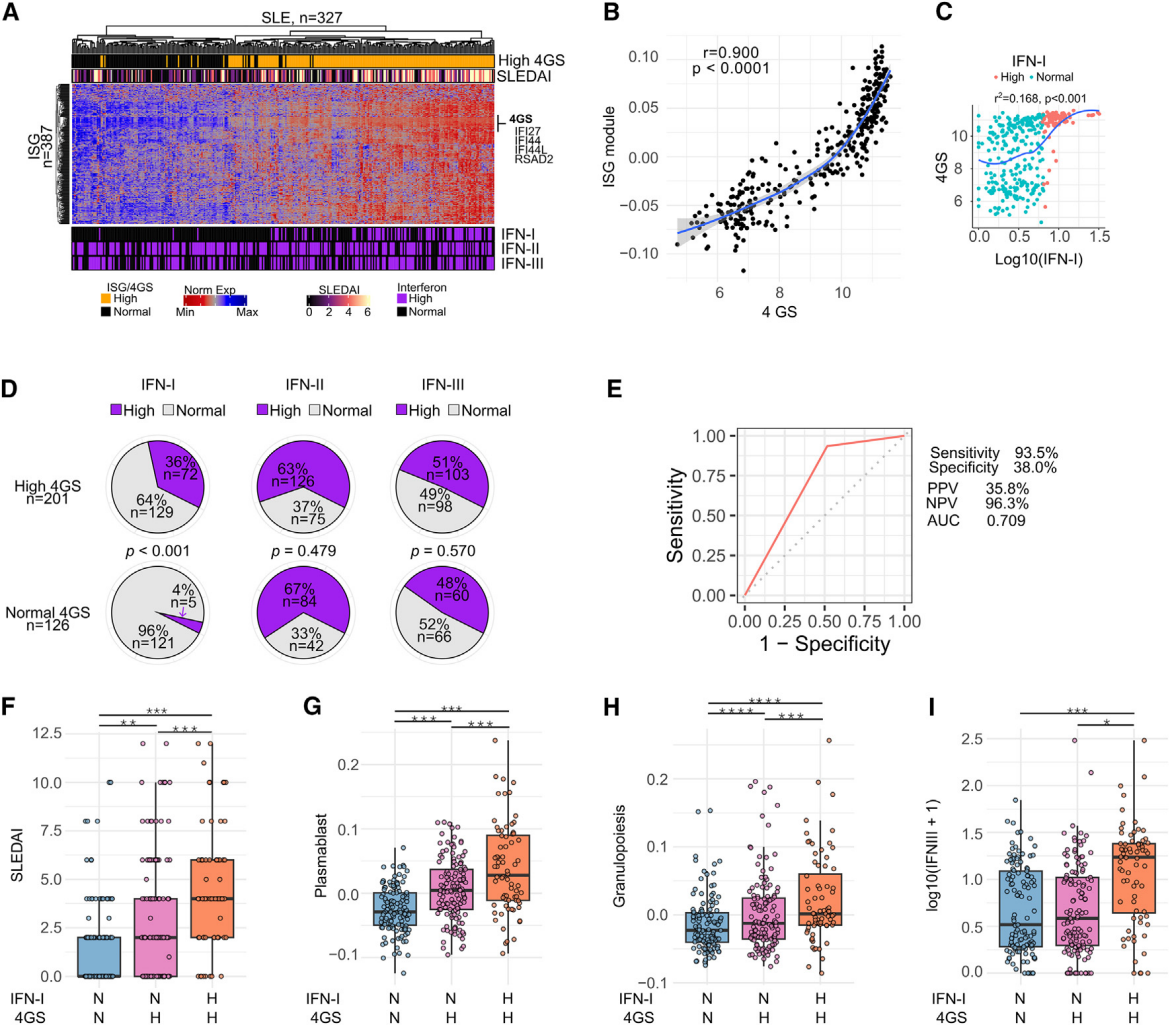
Relationship between IFN-induced gene expression and activity levels of IFNs (A) Hierarchical clustering of 387 transcripts from the ISG module defined in Figure 4G. Each column represents an individual patient and each row an individual gene. The top annotations represent the four-gene IFN signature (4GS) as a qualitative variable and SLEDAI score. The bottom annotations represent IFN activity levels as dichotomic variables. (B) Correlation (Person’s r) between the averaged 4GS and the ISG module. (C) Correlation between the averaged 4GS and log-transformed IFN-I activity. (D) Frequency of normal and increased levels of IFNs according to the 4GS. (E) ROC curve analysis of elevated 4GS as classifier of increased levels of IFN-I. (F‒H) Comparison of SLEDAI (F), plasmablast module (G), granulopoiesis module (H), and increased IFN-III activity (I) in patients with SLE according to increased IFN-I and 4GS (N = normal, H = high). IFN-I N + 4GS N (*n* = 121), IFN-I N + 4GS H (*n* = 129), IFN-I H + 4GS H (*n* = 72). Comparisons were done by using a linear mixed-effects linear model to account for subjects with repeated samples. In (F)–(I), patients with high IFN-I and normal 4GS were excluded from the analysis because of the small number in this group.

To better understand the relationship between the IFN signature and IFN types in SLE, we performed unsupervised hierarchical clustering using 387 transcripts from the ISG module (Figure 5A). Patients with SLE grouped in a gradient of increased expression of ISGs, which paralleled with disease activity and increased levels of IFN-I (Figure 5A). A common strategy used in clinical trials targeting the IFN-I pathway is to average the expression of four genes (IFI27, IFI44, IFI44L, and RSAD2) to classify patients with SLE according to IFN activity.^16^^,^^17^^,^^18^^,^^47^ Using the four-gene IFN signature (4GS), patients with SLE are classified as having a high or low IFN signature when their 4GS was higher or lower than the mean + 2 SD compared to healthy controls.^16^^,^^17^^,^^18^^,^^47^ Using this approach, we confirmed that the 4GS strongly correlates with the ISG module (r = 0.900, *p* < 0.0001) (Figure 5B). Paradoxically, however, the 4GS showed a poor correlation with IFN-I activity levels (r2 = 0.168, *p* < 0.001) (Figure 5C). This finding is explained because only 36% (72/201) of SLE samples with a high 4GS have increased activity levels of IFN-I (Figure 5D). Thus, although it is certain that increased levels of IFN-I are strongly associated with the IFN signature (72/77, 93.5%, *p* < 0.001), up to 64% of samples with a high 4GS contain IFN-I activity levels within a normal range. Indeed, the predictive value of the 4GS was highly sensitive to detect patients with SLE with high IFN-I activity (93%), but its specificity was low (38%). Similarly, the 4GS showed low positive and high negative predictive values (35.8% and 96.3%, respectively) to detect increased levels of IFN-I (Figure 5E). Importantly, increased activity levels of IFN-II and IFN-III were similar in samples with normal or elevated 4GSs (IFN-II, *p* = 0.479; IFN-III, *p* < 0.570) (Figure 5D), implying that neither IFN-II nor IFN-III can explain the expression of the IFN signature in patients with normal activity levels of IFN-I.

When we compared clinical and transcriptional features of patients with SLE according to the 4GS and IFN-I activity, we found that patients with increased 4GS and either normal or elevated IFN-I activity were characterized by higher SLEDAI, plasmablast, and granulopoiesis signatures compared to patients with normal 4GS expression. The presence of elevated 4GS and IFN-I levels, however, identified a patient subset with the highest clinical and transcriptional disease activity in SLE (Figures 5F–5H). Thus, the data support that, independently of the driver of the IFN signature, the expression of this transcriptional profile is associated with higher disease activity, which seems to be amplified by increased levels of IFN-I. Interestingly, the subset of patients with increased 4GSs and IFN-I activity also showed higher levels of IFN-III (Figure 5I), indicating that IFN-III may enhance IFN-induced gene expression when co-elevated with IFN-I. Patients with high IFN-I and normal 4GSs were excluded from the analysis due to the small number in this group.

## Discussion

IFNs are promising therapeutic targets in SLE. A deeper understanding of the clinical and pathogenic significance of IFNs in SLE has therefore critical implication for both diagnosis and treatment. To fill gaps in the relationship between IFNs and SLE, we used a unique and convenient assay to precisely measure the independent activity of each IFN type in a large and prospective SLE cohort with extensive clinical, laboratory, and whole-blood transcriptional data. Using this approach, we uncovered previously unknown insights into the potential role of IFNs in SLE pathogenesis and provided a rational explanation for the heterogeneous response in clinical trials targeting IFN-I in SLE. Overall, our data support an essential role of IFNs in SLE pathogenesis and disease activity but only in specific subsets of patients.

While there is no gold-standard assay for measuring IFNs in SLE, previous studies have shown that levels of IFN-I vary depending on disease activity, with high levels found in up to 80% of patients with active disease and 9% on samples obtained during disease quiescence.^48^ The prevalence of elevated IFN levels is thus expected to vary across real-life SLE cohorts. In SPARE, 32% of patients had increased IFN-I activity at the time of recruitment, with IFN-II and IFN-III elevated in 66% and 55% of patients, respectively. Interestingly, while increased activity levels of IFN-II and IFN-III were twice as common as increased levels of IFN-I in SLE, our data support IFN-I as the master regulator of disease activity in SLE, which is consistent with previous studies.^26^^,^^28^^,^^49^^,^^50^^,^^51^ Different from the current paradigm, however, our study indicates that disease activity features associated with IFN-I are determined by the co-elevation of the other IFN types. Thus, increased activity levels of IFN-I alone were strongly associated with cutaneous lupus, which explains why distinct therapies targeting the IFN-I pathway (either IFN-I-producing cells, soluble IFN-I, or the IFN-I receptor) showed the most efficacy in patients with SLE with cutaneous disease.^16^^,^^17^^,^^18^^,^^19^^,^^20^^,^^21^ In contrast, features linked to systemic disease activity previously attributed to IFN-I (such as arthritis, nephritis, and low complement)^49^^,^^52^ were associated with increased activity levels of IFN-I + IFN-II + IFN-III, indicating a synergistic effect of the three IFN families in this severe disease subset.

Interestingly, while individual analyses of IFN types showed clinical associations with elevated IFN-II and IFN-III, for instance, IFN-II with arthritis and IFN-III with SLEDAI and proteinuria, these associations were not confirmed in the analysis of the co-elevation of IFNs, demonstrating that the individual assessment of IFN types is not informative of clinical subsets in SLE. Indeed, in the absence of IFN-I, the elevation of IFN-II or IFN-II + IFN-III showed no association with any particular feature of disease activity, and only IFN-II + IFN-III was linked to a mild increase in SLEDAI. Thus, while increased levels of IFN-II and IFN-II + IFN-III are the most prevalent IFN types in SLE, the data point to a model in which the co-elevation of IFN-II and IFN-III creates a low disease activity environment that is amplified by the addition of IFN-I, acting as the determinant to evolve to a more severe disease. The finding that elevated IFN-II occurs years before SLE diagnosis, but the disease becomes clinically evident following the co-elevation of IFN-I, further supports this model.^53^ In the absence of other IFN types, however, increased activity levels of IFN-I were only associated with cutaneous disease. The finding that hallmark autoantibodies linked to SLE pathogenesis were only associated with disease subsets with high IFN-I activity, as well as the unique association of IFN-I with transcriptional fingerprints linked to disease activity (plasmablasts and granulopoiesis), further supports the notion that IFN-I is the main IFN type that dictates the disease outcome in a subset of patients with SLE. Nevertheless, the data also anticipate that blocking the IFN-I pathway will be insufficient to treat clinical subsets driven by the combined effect of the three IFN types.

In contrast to the notion that IFN-I levels (as determined indirectly by quantification of IFN-I-induced chemokines and ISG expression) do not fluctuate with changes in SLE disease activity,^52^^,^^54^^,^^55^ we found that IFN-I activity levels correlate with changes in SLEDAI, anti-dsDNA, and C3, implying a potential disconnect between IFN-I levels and biomarkers attributed to the activity of IFN-I. Indeed, whereas increased activity levels of IFN-I were strikingly associated with the IFN signature, we found that the IFN signature was not associated with increased levels of IFN-I in up to 64% of the cases. This finding offers a rational explanation for why blocking the IFN-I receptor showed efficacy only in a subset of patients with SLE with a high IFN signature^18^ and highlights that anti-IFN-I therapies are more likely to be effective in patients with high levels of IFN-I rather than a high IFN signature.

Importantly, the lack of correlation between increased IFN-I activity and the IFN signature is not explained by the sensitivity of our assay to detect IFN-I. Indeed, IFN-I activity was detected in nearly all samples from patients with SLE and healthy controls. However, increased IFN-I activity was found in only 32% of patients with SLE, with IFN-I levels in 68% of patients falling within the range of healthy controls, which corresponds to the constitutive production of IFN-I.^56^ Notably, despite differences in the approaches to measure IFN-I, the lower detection limit of IFN-I by HEK-Blue IFN-α/β reporter cells is similar to other assays used to measure IFN-α protein and IFN-I activity (i.e., 1 IU/mL), such as the dissociation-enhanced lanthanide fluoroimmunoassay and the WISH assay.^36^^,^^50^^,^^57^^,^^58^^,^^59^ Thus, our detection system is as sensitive as other commonly used assays while also being IFN-type specific, simpler, and requiring less serum (the WISH assay, for example, requires 50% serum compared to 20% serum when using HEK-Blue IFN-α/β reporter cells).^36^

To improve the sensitivity of IFN-α detection in SLE, attomolar (femtograms [fg] per milliliter) concentrations have been measured using Simoa digital ELISA, with an IFN-α detection level of less than 0.1 fg/mL.^27^^,^^28^ However, despite the high sensitivity of detection, increased levels of IFN-α in the range of 10–300 fg/mL showed no difference in SLE disease activity compared to IFN-α levels below 10 fg/mL. An association with active SLE was only observed in patients with IFN-α levels above ∼300 fg/mL.^27^^,^^28^ It is worth noting that an efficient biological response induced by IFN-α, as measured by a cytopathic assay, was only achieved at concentrations greater than 300 fg/mL (equivalent to 10 IU/mL),^27^ which is within the detection limit of our functional assay (1 IU/mL). Therefore, the Simoa digital ELISA data indicate that measuring small amounts of IFN-α, which have no significant effect on cellular activation, has no value as a marker of disease activity in SLE.^27^^,^^28^ Moreover, these data support that the IFN-I activity levels detected in our study, which are below the functional effect of 300 fg/mL, are useful to identify patients in whom IFN-I levels have a biological effect on disease activity in SLE.

Because the IFN signature and high levels of IFN-I are more likely to co-exist in a subset of patients with high disease activity, the association between the IFN signature and increased IFN-I is expected to vary across cohorts depending on the proportion of patients with different levels of disease activity. For instance, a recent study showed a striking correlation between IFN-α levels and the IFN signature in an SLE cohort with higher disease activity (SLEDAI range: 0–42)^49^ compared to the SPARE cohort (SLEDAI range: 0–15). Since patients expressing the IFN signature with normal levels of IFN-I are found in the group of mild disease activity, which is enriched in the SPARE cohort, these patients are likely to go unnoticed or be considered outliers in cohorts lacking in this specific clinical subset of patients. Importantly, increased levels of neither IFN-II nor IFN-III explain the expression of the IFN signature in the subset of patients with normal levels of IFN-I. Consistent with this finding, a recent study showed no correlation between IFN-II and ISG expression in SLE.^49^

Why patients with SLE overexpress ISGs while having normal IFN-I levels is unclear. It is possible that a subset of patients with SLE are hypersensitive to IFN-I. Studies to support this notion, however, are missing. Although there is evidence that lupus keratinocytes are hyperresponsive to high amounts of IFN-I (1000 IU/mL),^60^ studies using IFN-I within a normal range (<5.17 IU/mL) are lacking. Alternatively, it is possible that the IFN signature is induced by mechanisms other than IFN-I, for instance, by other cytokines. Indeed, not every feature in SLE, such as cytopenia, serositis, vasculitis, and APS, was associated with increased activity levels of IFNs, supporting the notion of IFN-independent endotypes in SLE.

While our study and others^49^ found that IFN-II plays no role in the IFN signature, as well as disease activity and autoantibodies, and that it has been shown to be an ineffective therapeutic target in SLE,^61^^,^^62^ our data suggest that increased levels of IFN-II are associated with IFN-I-independent transcriptional profiles dysregulated in SLE, including mitochondria, oxidative phosphorylation, and the clonal expansion of CD8^+^*GZMH*^*+*^ cells.^7^^,^^8^ Moreover, IFN-III seems to enhance the IFN signature when co-elevated with IFN-I. The co-activation of IFN-I-dependent and IFN-I-independent immune pathways explains how the three IFN types work synergistically in severe SLE.

In summary, our findings underscore that both IFN-dependent and IFN-independent pathways contribute to clinical heterogeneity in SLE and that anti-IFN-I therapies are more likely to be beneficial in patients with high levels of bioactive IFN-I rather than a high IFN signature.

### Limitations of the study

We acknowledge that our data have limitations to show the influence of treatment on IFN levels. Patients were not followed at regular time intervals, and the number of patients with at least three visits (*n* = 43) would not have enough power to model the effect of the different treatments and their interactions with the IFN subsets. Instead, we decided to model the probability of a patient with different treatments to present with either single or combined elevation of IFN types at the clinical visits compared to normal IFN. Similarly, we do not have longitudinal measurements of all autoantibodies to analyze the correlation between changes of autoantibody titers and the IFN levels, except for anti-dsDNA.

## STAR★Methods

### Key resources table

**Table:** 

REAGENT or RESOURCE	SOURCE	IDENTIFIER
**Biological samples**		

SLE and control plasma or serum samples	Patients and healthy control subjects	N/A

**Critica commercia assays**		

HEK-BlueTM IFN-α/β cells	InvivoGen	Cat# hkb-ifnab; RRID: CVCL_KT26
HEK-Blue™ IFN-γ cells	InvivoGen	Cat# hkb-ifng; RRID: CVCL_UF30
HEK-Blue™ IFN-λ cells	InvivoGen	Cat# hkb-ifnl
QUANTI-Blue™	InvivoGen	Cat# rep-qb1
IFN-α6	PBL	Cat# 11165
IFN-γ	PeproTech	Cat# 300-02
IFN-λ3 (IL-28B)	Gibco	Cat# PHC0894

**Deposited data**		

Whole blood gene expression data by microarray	Gene expression Omnibus	GSE45291; GSE121239

**Softwareand algorithms**		

Oligo	https://bioconductor.org/packages/release/bioc/html/oligo.html	Ver. 1.6.6
Genefilter	https://bioconductor.org/packages/release/bioc/html/genefilter.html	Ver. 1.84
Limma	https://bioconductor.org/packages/release/bioc/html/limma.html	Ver. 3.58.1
Metascape.org	https://metascape.org/	v3.5.20240101
Complexheatmap	https://bioconductor.org/packages/devel/bioc/html/ComplexHeatmap.html	Ver. 2.19.0
WGCNA	https://cran.r-project.org/web/packages/WGCNA/index.html	Ver. 1.72–5
Cytoscape	https://cytoscape.org/	Ver. 3.10.2
STRING	https://apps.cytoscape.org/apps/stringapp	Ver. 2.0.3
rptR	https://github.com/mastoffel/rptR	Ver.0.9.1
Brms	https://github.com/paul-buerkner/brms/	Ver.2.21.0
Lme4	https://github.com/lme4/lme4	Ver. 1.1–14
nnet	https://cran.r-project.org/web/packages/nnet/index.html	Ver. 7.3–19

### Resource availability

#### Lead contact

Further information and requests for resources and reagents should be directed to and will be fulfilled by the Lead contact, Dr. Eduardo Gómez-Bañuelos (jgomezb1@jhmi.edu).

#### Materials availability

There is no availability of biological material because we utilized unique patient samples. This study did not generate new unique reagents. The IFN reporter cell lines are commercially available from InvivoGen.

#### Data and code availability

Microarray data are available from the Gene Expression Omnibus under accession number GSE45291 and GSE121239. Any additional information required to reanalyze the data reported in this work paper is available from the lead contact upon request. This paper does not report original code.

### Experimental model and study participants details

#### Study design

The objective of this study was to investigate the relationship between IFN types and clinical and transcriptional profiles in SLE. To reach this goal, we used a cell-based reporter assay that precisely quantifies activity levels of IFN-I, IFN-II and IFN-III in combination with clinical and whole blood transcriptional data from a large prospective cohort of patients with SLE. We studied 341 plasma/serum samples from 191 SLE patients from the “Study of biological Pathways, Disease Activity and Response markers in patients with Systemic Lupus Erythematosus” (SPARE), and 56 serum samples from healthy controls. In addition, plasma and serum were collected in parallel from 20 consecutive patients with SLE. SPARE is a prospective observational cohort that has been extensively described previously.^41^^,^^42^ Adult patients (age 18 to 75 years-old) who met the definition of SLE per the revised American College of Rheumatology classification criteria^63^ were eligible into the study. Demographic characteristics of participants are shown in Table S1. At baseline, the patient’s medical history was reviewed, and information on current medications was recorded. The SPARE cohort patients were followed-up over a 2-year period. All patients were treated according to standard clinical practice. Disease activity was assessed using the Safety of Estrogens in Lupus Erythematosus: National Assessment (SELENA) version of the Systemic Lupus Erythematosus Disease Activity Index (SLEDAI)^64^ and physician global assessment (PGA).^65^ C3, C4, anti-dsDNA (Crithidia), complete blood cell count and urinalysis were performed at every visit. All samples were obtained under informed written consent approved by the Johns Hopkins University Institutional Review Board.

### Method details

#### Quantification of activity levels of IFNs

Activity levels of IFN-I, IFN-II and IFN-III were determined in serum/plasma using the HEK-Blue IFN-α/β (Cat. # hkb-ifnab), HEK-Blue IFN-γ (Cat. # hkb-ifng) and HEK-Blue IFN-λ cells (Cat. # hkb-ifnl) from InvivoGen, respectively. Upon activation with IFN, the reporter cells secrete embryonic alkaline phosphatase (SEAP) under the control of the ISG54 promoter. Thus, using a colorimetric substrate, SEAP levels are used to quantify IFN-induced activation in 96-well plates. Briefly, each cell line was plated in 96 well plates and incubated with 100 μL DMEM containing 20% SLE or healthy control heparin/citrate plasma or serum in duplicated. Since recalcification of citrated plasma in DMEM activates the coagulation pathway, 0.6 IU of sodium heparin was added per 100 μL media. To control for heparin, assays performed in serum also contained 0.6 IU of sodium heparin. After 24 h at 37°C, 20 μL of supernatant were incubated at room temperature with 180 μL of QUANTI-Blue alkaline phosphatase substrate (InvivoGen) for 2 h to determine the activity of IFN-I and IFN-III, and for 18 h for IFN-II activity. Using this approach, we found that activity levels of IFNs were similar in serum and plasma collected in parallel (Figure S1). To quantify the IFN activity, we interpolated the 620 nm absorbance to a standard curve made with serial dilutions of human recombinant IFN-α6 (PBL, Cat. # 11165), IFN-γ (PeproTech, Cat. # 300-02), and IFN-λ3 (IL-28B) (Gibco, Cat. # PHC0894). Cutoff values for IFN-I and IFN-II were determined from healthy control IFN activity using ROC curve analyses setting specificity of 95%. Cutoff value for IFN-III was determined by using a cutoff point above the 95^th^ percentile of IFN-III activity in healthy controls.

#### Differential gene expression analyses

Gene expression analysis from the SPARE cohort was previously described.^42^ CEL files were subjected to RMA background correction, and quantile normalization, using the Oligo package.^2^ To select only expressed genes in whole blood, we filtered out transcripts that had a raw signal <100 in less than 10% of samples with the genefilter R package. All calculations and analyses were performed using R (ver 4.0.2) and Bioconductor (ver 3.13).^66^ Differentially expressed transcripts (DETs) were analyzed fitting a mixed-effects linear model adjusted by anti-dsDNA and SLEDAI using the duplicated correlation function in the R package limma.^67^ Each IFN group was tested against the IFN normal group. We determined the adjusted *p* value of each transcript using the function *‘decideTests’*, with the ‘global’ method to compare the number of DET found for each comparison. DET was considered when the adjusted *p* value was <0.05. To increase the statistical power and reduce the false discovery rate of the differential gene expression analysis, we only included the 7500 most variable genes among the samples. Functional gene set enrichment analyses were carried out using the online server Metascape.org, uploading the DET of each subset in a multi-list, in order to compare the enrichment between IFN subgroups using the metanalysis function of Metascape.^68^ Heatmap visualization of the Top 100 enriched pathways between each IFN subgroup was done using the Complexheatmap R package.^69^

#### Gene co-expression modules

Gene co-expression modules were determined by weighted correlation network analysis (WGCNA), implemented with the WGCNA R package.^46^ The weighted network was constructed using a thresholding power β of 10 based on the criterion of approximate scale-free topology, a minimum module size of 30, and a medium sensitivity for cluster splitting. Module eigengenes (first principal component) were used as a measure of module activity. Modules were interpreted by gene set enrichment analysis using Metascape.org.^68^ To interpret the biological significance of the WGCNA modules, we performed enrichment analysis on the online platform Metascape.org using the express analysis options. All modules went through different rounds of pathway selection to interpret their significance. First, we considered the top 20 summary terms along with their clustered member terms. To simplify the enrichment, we selected only the terms with an adjusted *p* value (q-value) < 0.01 and selected the top 10 terms with the highest ratio of present genes within the module/genes within the term. Next, to verify the enrichment and refine the module annotation, we performed a network analysis of each module using Cytoscape and STRING. Then, we examined the largest connected component of the network. If this component pointed toward genes characteristic of specific cell types, we refined the modules annotation of the module by considering the cell signature enrichment of Metascape. When modules were composed by a large (>100 nodes) and densely connected network, we simplified the network by identifying the top 3 hub genes and performed enrichment analysis on their subnetworks.^70^^,^^71^^,^^72^

### Quantification and statistical analysis

#### Statistical analyses

Comparisons of continuous variables between groups were done using Student’s T test and ANOVA test as indicated. Fisher’s exact test and χ^2^ tests were used for univariate analysis on SPARE cohort variables, as appropriate. Correlations between variables were estimated using Pearsons’s r correlation coefficient. The repeatability or intraclass correlation coefficients (ICC), and their corresponding 95% confidence intervals (95% CI), for the IFN types, bodyweight and SLEDAI were estimated using a mixed-effects model implemented with the rptR package.^44^ To estimate the between-patient correlation of disease activity parameters and the IFN types we fitted a Bayesian multivariate multilevel model considering both the IFN type and disease activity as outcomes, and patients with repeated samples as random effects, using the following formula mvbind (y, x) ∼ 1 + (1|c|patient), data = data) using brms.^73^ To control for the effect of subjects with longitudinal samples, associations between clinical variables or module activity with the continuous levels of the IFN types were determined by fitting mixed-effects models using the lme4 package with the formula *y ∼ x + disease duration + (1|Patient ID)*.^74^ Associations between categorical variables were determined by fitting multinomial log-linear models via neural networks considering patients with repeated samples as a random effect using the R package nnet with the formula *y ∼ x + disease duration + (1|Patient ID)*.^75^ Statistical significance was set at *p* < 0.05. The statistical analyses were carried out with the R software version 4.0.2.

## Supplemental information

Document S1. Figures S1 and Tables S1‒S6

Data S1. Differentially expressed transcripts between individual and combined IFN types in SLE, related to Figure 4 Differentially expressed transcripts (DET) in SLE visits with single elevated IFN-I (A, *n* = 20), IFN-I + II + III (B, *n* = 48), single elevated IFN-II (C, *n* = 63), and IFN-II + III (D, *n* = 94) vs. normal IFN (*n* = 75). Differential expression analysis was done using a linear mixed-effects model to account for patients with repeated samples. DET were determined when the adjusted *p* value was <0.05. D. Enriched pathways associated with IFN groups. Enrichment was performed using the multi-gene-list meta-analysis feature from Metascape.org. The gene lists include the DET from A–D.

Data S2. Weighted correlation network analysis-defined modules, related to Figure 4 A. WGCNA module description including most representative biological theme, designated color from WGCNA output, number and included genes. B–Y. Top 10 most significant enriched pathways of each module along with the STRING network view of the largest connected component. Top 3 hub genes are shown in red, yellow and orange.

Document S2. Article plus supplemental information

## References

[1] Rahman A., Isenberg D.A. (2008). Systemic lupus erythematosus. N. Engl. J. Med..

[2] Banchereau R., Hong S., Cantarel B., Baldwin N., Baisch J., Edens M., Cepika A.M., Acs P., Turner J., Anguiano E. (2016). Personalized Immunomonitoring Uncovers Molecular Networks that Stratify Lupus Patients. Cell.

[3] Nehar-Belaid D., Hong S., Marches R., Chen G., Bolisetty M., Baisch J., Walters L., Punaro M., Rossi R.J., Chung C.H. (2020). Mapping systemic lupus erythematosus heterogeneity at the single-cell level. Nat. Immunol..

[4] Bennett L., Palucka A.K., Arce E., Cantrell V., Borvak J., Banchereau J., Pascual V. (2003). Interferon and granulopoiesis signatures in systemic lupus erythematosus blood. J. Exp. Med..

[5] Baechler E.C., Batliwalla F.M., Karypis G., Gaffney P.M., Ortmann W.A., Espe K.J., Shark K.B., Grande W.J., Hughes K.M., Kapur V. (2003). Interferon-inducible gene expression signature in peripheral blood cells of patients with severe lupus. Proc. Natl. Acad. Sci. USA.

[6] Arazi A., Rao D.A., Berthier C.C., Davidson A., Liu Y., Hoover P.J., Chicoine A., Eisenhaure T.M., Jonsson A.H., Li S. (2019). The immune cell landscape in kidneys of patients with lupus nephritis. Nat. Immunol..

[7] Perez R.K., Gordon M.G., Subramaniam M., Kim M.C., Hartoularos G.C., Targ S., Sun Y., Ogorodnikov A., Bueno R., Lu A. (2022). Single-cell RNA-seq reveals cell type-specific molecular and genetic associations to lupus. Science.

[8] Nakano M., Ota M., Takeshima Y., Iwasaki Y., Hatano H., Nagafuchi Y., Itamiya T., Maeda J., Yoshida R., Yamada S. (2022). Distinct transcriptome architectures underlying lupus establishment and exacerbation. Cell.

[9] Billi A.C., Ma F., Plazyo O., Gharaee-Kermani M., Wasikowski R., Hile G.A., Xing X., Yee C.M., Rizvi S.M., Maz M.P. (2022). Nonlesional lupus skin contributes to inflammatory education of myeloid cells and primes for cutaneous inflammation. Sci. Transl. Med..

[10] Der E., Suryawanshi H., Morozov P., Kustagi M., Goilav B., Ranabothu S., Izmirly P., Clancy R., Belmont H.M., Koenigsberg M. (2019). Tubular cell and keratinocyte single-cell transcriptomics applied to lupus nephritis reveal type I IFN and fibrosis relevant pathways. Nat. Immunol..

[11] Walter M.R. (2020). The Role of Structure in the Biology of Interferon Signaling. Front. Immunol..

[12] Caielli S., Wan Z., Pascual V. (2023). Systemic Lupus Erythematosus Pathogenesis: Interferon and Beyond. Annu. Rev. Immunol..

[13] Schneider W.M., Chevillotte M.D., Rice C.M. (2014). Interferon-stimulated genes: a complex web of host defenses. Annu. Rev. Immunol..

[14] El-Sherbiny Y.M., Psarras A., Md Yusof M.Y., Hensor E.M.A., Tooze R., Doody G., Mohamed A.A.A., McGonagle D., Wittmann M., Emery P., Vital E.M. (2018). A novel two-score system for interferon status segregates autoimmune diseases and correlates with clinical features. Sci. Rep..

[15] Niewold T.B. (2011). Interferon Alpha as a Primary Pathogenic Factor in Human Lupus. J. Interferon Cytokine Res..

[16] Furie R.A., Morand E.F., Bruce I.N., Manzi S., Kalunian K.C., Vital E.M., Lawrence Ford T., Gupta R., Hiepe F., Santiago M. (2019). Type I interferon inhibitor anifrolumab in active systemic lupus erythematosus (TULIP-1) a randomised, controlled, phase 3 trial. Lancet. Rheumatol..

[17] Khamashta M., Merrill J.T., Werth V.P., Furie R., Kalunian K., Illei G.G., Drappa J., Wang L., Greth W., CD1067 study investigators (2016). Sifalimumab, an anti-interferon-alpha monoclonal antibody, in moderate to severe systemic lupus erythematosus: a randomised, double-blind, placebo-controlled study. Ann. Rheum. Dis..

[18] Morand E.F., Furie R., Tanaka Y., Bruce I.N., Askanase A.D., Richez C., Bae S.C., Brohawn P.Z., Pineda L., Berglind A. (2020). Trial of Anifrolumab in Active Systemic Lupus Erythematosus. N. Engl. J. Med..

[19] Werth V.P., Furie R.A., Romero-Diaz J., Navarra S., Kalunian K., van Vollenhoven R.F., Nyberg F., Kaffenberger B.H., Sheikh S.Z., Radunovic G. (2022). Trial of Anti-BDCA2 Antibody Litifilimab for Cutaneous Lupus Erythematosus. N. Engl. J. Med..

[20] Furie R., Werth V.P., Merola J.F., Stevenson L., Reynolds T.L., Naik H., Wang W., Christmann R., Gardet A., Pellerin A. (2019). Monoclonal antibody targeting BDCA2 ameliorates skin lesions in systemic lupus erythematosus. J. Clin. Invest..

[21] Karnell J.L., Wu Y., Mittereder N., Smith M.A., Gunsior M., Yan L., Casey K.A., Henault J., Riggs J.M., Nicholson S.M. (2021). Depleting plasmacytoid dendritic cells reduces local type I interferon responses and disease activity in patients with cutaneous lupus. Sci. Transl. Med..

[22] Paredes J.L., Niewold T.B. (2020). Type I interferon antagonists in clinical development for lupus. Expert Opin. Investig. Drugs.

[23] Hooks J.J., Moutsopoulos H.M., Geis S.A., Stahl N.I., Decker J.L., Notkins A.L. (1979). Immune interferon in the circulation of patients with autoimmune disease. N. Engl. J. Med..

[24] Goel R.R., Kotenko S.V., Kaplan M.J. (2021). Interferon lambda in inflammation and autoimmune rheumatic diseases. Nat. Rev. Rheumatol..

[25] Liu W., Zhang S., Wang J. (2022). IFN-gamma, should not be ignored in SLE. Front. Immunol..

[26] Oke V., Gunnarsson I., Dorschner J., Eketjäll S., Zickert A., Niewold T.B., Svenungsson E. (2019). High levels of circulating interferons type I, type II and type III associate with distinct clinical features of active systemic lupus erythematosus. Arthritis Res. Ther..

[27] Rodero M.P., Decalf J., Bondet V., Hunt D., Rice G.I., Werneke S., McGlasson S.L., Alyanakian M.A., Bader-Meunier B., Barnerias C. (2017). Detection of interferon alpha protein reveals differential levels and cellular sources in disease. J. Exp. Med..

[28] Mathian A., Mouries-Martin S., Dorgham K., Devilliers H., Barnabei L., Ben Salah E., Cohen-Aubart F., Garrido Castillo L., Haroche J., Hie M. (2019). Monitoring Disease Activity in Systemic Lupus Erythematosus With Single-Molecule Array Digital Enzyme-Linked Immunosorbent Assay Quantification of Serum Interferon-alpha. Arthritis Rheumatol..

[29] Moll H.P., Maier T., Zommer A., Lavoie T., Brostjan C. (2011). The differential activity of interferon-alpha subtypes is consistent among distinct target genes and cell types. Cytokine.

[30] Jabs W.J., Hennig C., Zawatzky R., Kirchner H. (1999). Failure to detect antiviral activity in serum and plasma of healthy individuals displaying high activity in ELISA for IFN-alpha and IFN-beta. J. Interferon Cytokine Res..

[31] Wu Q., Yang Q., Lourenco E., Sun H., Zhang Y. (2011). Interferon-lambda1 induces peripheral blood mononuclear cell-derived chemokines secretion in patients with systemic lupus erythematosus: its correlation with disease activity. Arthritis Res. Ther..

[32] Amezcua-Guerra L.M., Márquez-Velasco R., Chávez-Rueda A.K., Castillo-Martínez D., Massó F., Páez A., Colín-Fuentes J., Bojalil R. (2017). Type III Interferons in Systemic Lupus Erythematosus: Association Between Interferon lambda3, Disease Activity, and Anti-Ro/SSA Antibodies. J. Clin. Rheumatol..

[33] Chen J.Y., Wang C.M., Chen T.D., Jan Wu Y.J., Lin J.C., Lu L.Y., Wu J. (2018). Interferon-lambda3/4 genetic variants and interferon-lambda3 serum levels are biomarkers of lupus nephritis and disease activity in Taiwanese. Arthritis Res. Ther..

[34] Zickert A., Oke V., Parodis I., Svenungsson E., Sundström Y., Gunnarsson I. (2016). Interferon (IFN)-lambda is a potential mediator in lupus nephritis. Lupus Sci. Med..

[35] Adel Y., Sadeq Y. (2020). Impact of IL-34, IFN-alpha and IFN-lambda1 on activity of systemic lupus erythematosus in Egyptian patients. Reumatologia.

[36] Hua J., Kirou K., Lee C., Crow M.K. (2006). Functional assay of type I interferon in systemic lupus erythematosus plasma and association with anti-RNA binding protein autoantibodies. Arthritis Rheum..

[37] Pestka S., Krause C.D., Walter M.R. (2004). Interferons, interferon-like cytokines, and their receptors. Immunol. Rev..

[38] Preble O.T., Black R.J., Friedman R.M., Klippel J.H., Vilcek J. (1982). Systemic lupus erythematosus: presence in human serum of an unusual acid-labile leukocyte interferon. Science.

[39] Niewold T.B., Hua J., Lehman T.J.A., Harley J.B., Crow M.K. (2007). High serum IFN-alpha activity is a heritable risk factor for systemic lupus erythematosus. Genes Immun..

[40] Harley I.T., Niewold T.B., Stormont R.M., Kaufman K.M., Glenn S.B., Franek B.S., Kelly J.A., Kilpatrick J.R., Hutchings D., Divers J. (2010). The role of genetic variation near interferon-kappa in systemic lupus erythematosus. J. Biomed. Biotechnol..

[41] Zollars E., Bienkowska J., Czerkowicz J., Allaire N., Ranger A.M., Magder L., Petri M. (2015). BAFF (B cell activating factor) transcript level in peripheral blood of patients with SLE is associated with same-day disease activity as well as global activity over the next year. Lupus Sci. Med..

[42] Zollars E., Courtney S.M., Wolf B.J., Allaire N., Ranger A., Hardiman G., Petri M. (2016). Clinical Application of a Modular Genomics Technique in Systemic Lupus Erythematosus: Progress towards Precision Medicine. Int. J. Genomics.

[43] Toro-Domínguez D., Martorell-Marugán J., Goldman D., Petri M., Carmona-Sáez P., Alarcón-Riquelme M.E. (2018). Stratification of Systemic Lupus Erythematosus Patients Into Three Groups of Disease Activity Progression According to Longitudinal Gene Expression. Arthritis Rheumatol..

[44] Stoffel M.A., Nakagawa S., Schielzeth H. (2017). rptR: repeatability estimation and variance decomposition by generalized linear mixed-effects models. Methods Ecol. Evol..

[45] Gomez-Bañuelos E., Wahadat M.J., Li J., Paz M., Antiochos B., Celia A.I., Andrade V., Ferris D.P., Goldman D.W., Darrah E. (2022). Alternative exon usage in TRIM21 determines the antigenicity of Ro52/TRIM21 in systemic lupus erythematosus. JCI Insight.

[46] Langfelder P., Horvath S. (2008). WGCNA: an R package for weighted correlation network analysis. BMC Bioinf..

[47] Furie R., Khamashta M., Merrill J.T., Werth V.P., Kalunian K., Brohawn P., Illei G.G., Drappa J., Wang L., Yoo S., CD1013 Study Investigators (2017). Anifrolumab, an Anti-Interferon-alpha Receptor Monoclonal Antibody, in Moderate-to-Severe Systemic Lupus Erythematosus. Arthritis Rheumatol..

[48] Ytterberg S.R., Schnitzer T.J. (1982). Serum Interferon Levels in Patients with Systemic Lupus-Erythematosus. Arthritis Rheum..

[49] Chasset F., Mathian A., Dorgham K., Ribi C., Trendelenburg M., Huynh-Do U., Roux-Lombard P., Courvoisier D.S., Amoura Z., Gorochov G., Chizzolini C. (2022). Serum interferon-alpha levels and IFN type I-stimulated genes score perform equally to assess systemic lupus erythematosus disease activity. Ann. Rheum. Dis..

[50] Bengtsson A.A., Sturfelt G., Truedsson L., Blomberg J., Alm G., Vallin H., Rönnblom L. (2000). Activation of type I interferon system in systemic lupus erythematosus correlates with disease activity but not with antiretroviral antibodies. Lupus.

[51] Kirou K.A., Lee C., George S., Louca K., Peterson M.G.E., Crow M.K. (2005). Activation of the interferon-alpha pathway identifies a subgroup of systemic lupus erythematosus patients with distinct serologic features and active disease. Arthritis Rheum..

[52] Postal M., Vivaldo J.F., Fernandez-Ruiz R., Paredes J.L., Appenzeller S., Niewold T.B. (2020). Type I interferon in the pathogenesis of systemic lupus erythematosus. Curr. Opin. Immunol..

[53] Munroe M.E., Lu R., Zhao Y.D., Fife D.A., Robertson J.M., Guthridge J.M., Niewold T.B., Tsokos G.C., Keith M.P., Harley J.B., James J.A. (2016). Altered type II interferon precedes autoantibody accrual and elevated type I interferon activity prior to systemic lupus erythematosus classification. Ann. Rheum. Dis..

[54] Connelly K.L., Kandane-Rathnayake R., Huq M., Hoi A., Nikpour M., Morand E.F. (2018). Longitudinal association of type 1 interferon-induced chemokines with disease activity in systemic lupus erythematosus. Sci. Rep..

[55] Petri M., Singh S., Tesfasyone H., Dedrick R., Fry K., Lal P., Williams G., Bauer J., Gregersen P., Behrens T., Baechler E. (2009). Longitudinal expression of type I interferon responsive genes in systemic lupus erythematosus. Lupus.

[56] Gough D.J., Messina N.L., Clarke C.J.P., Johnstone R.W., Levy D.E. (2012). Constitutive type I interferon modulates homeostatic balance through tonic signaling. Immunity.

[57] Cederblad B., Blomberg S., Vallin H., Perers A., Alm G.V., Rönnblom L. (1998). Patients with systemic lupus erythematosus have reduced numbers of circulating natural interferon-alpha- producing cells. J. Autoimmun..

[58] Enocsson H., Wetterö J., Eloranta M.L., Gullstrand B., Svanberg C., Larsson M., Bengtsson A.A., Rönnblom L., Sjöwall C. (2021). Comparison of Surrogate Markers of the Type I Interferon Response and Their Ability to Mirror Disease Activity in Systemic Lupus Erythematosus. Front. Immunol..

[59] Balboni I., Niewold T.B., Morgan G., Limb C., Eloranta M.L., Rönnblom L., Utz P.J., Pachman L.M. (2013). Interferon-alpha induction and detection of anti-ro, anti-la, anti-sm, and anti-rnp autoantibodies by autoantigen microarray analysis in juvenile dermatomyositis. Arthritis Rheum..

[60] Tsoi L.C., Hile G.A., Berthier C.C., Sarkar M.K., Reed T.J., Liu J., Uppala R., Patrick M., Raja K., Xing X. (2019). Hypersensitive IFN Responses in Lupus Keratinocytes Reveal Key Mechanistic Determinants in Cutaneous Lupus. J. Immunol..

[61] Werth V.P., Fiorentino D., Sullivan B.A., Boedigheimer M.J., Chiu K., Wang C., Arnold G.E., Damore M.A., Bigler J., Welcher A.A. (2017). Brief Report: Pharmacodynamics, Safety, and Clinical Efficacy of AMG 811, a Human Anti-Interferon-gamma Antibody, in Patients With Discoid Lupus Erythematosus. Arthritis Rheumatol..

[62] Boedigheimer M.J., Martin D.A., Amoura Z., Sánchez-Guerrero J., Romero-Diaz J., Kivitz A., Aranow C., Chan T.M., Chong Y.B., Chiu K. (2017). Safety, pharmacokinetics and pharmacodynamics of AMG 811, an anti-interferon-gamma monoclonal antibody, in SLE subjects without or with lupus nephritis. Lupus Sci. Med..

[63] Hochberg M.C. (1997). Updating the American College of Rheumatology revised criteria for the classification of systemic lupus erythematosus. Arthritis Rheum..

[64] Petri M., Kim M.Y., Kalunian K.C., Grossman J., Hahn B.H., Sammaritano L.R., Lockshin M., merrill J.T., Belmont H.M., Askanase A.D. (2005). Combined oral contraceptives in women with systemic lupus erythematosus. N. Engl. J. Med..

[65] Petri M., Hellmann D., Hochberg M. (1992). Validity and reliability of lupus activity measures in the routine clinic setting. J. Rheumatol..

[66] Huber W., Carey V.J., Gentleman R., Anders S., Carlson M., Carvalho B.S., Bravo H.C., Davis S., Gatto L., Girke T. (2015). Orchestrating high-throughput genomic analysis with Bioconductor. Nat. Methods.

[67] Hoffman G.E., Roussos P. (2021). Dream: powerful differential expression analysis for repeated measures designs. Bioinformatics.

[68] Zhou Y., Zhou B., Pache L., Chang M., Khodabakhshi A.H., Tanaseichuk O., Benner C., Chanda S.K. (2019). Metascape provides a biologist-oriented resource for the analysis of systems-level datasets. Nat. Commun..

[69] Gu Z., Eils R., Schlesner M. (2016). Complex heatmaps reveal patterns and correlations in multidimensional genomic data. Bioinformatics.

[70] Shannon P., Markiel A., Ozier O., Baliga N.S., Wang J.T., Ramage D., Amin N., Schwikowski B., Ideker T. (2003). Cytoscape: a software environment for integrated models of biomolecular interaction networks. Genome Res..

[71] Doncheva N.T., Morris J.H., Gorodkin J., Jensen L.J. (2019). Cytoscape StringApp: Network Analysis and Visualization of Proteomics Data. J. Proteome Res..

[72] Chin C.H., Chen S.H., Wu H.H., Ho C.W., Ko M.T., Lin C.Y. (2014). cytoHubba: identifying hub objects and sub-networks from complex interactome. BMC Syst. Biol..

[73] Bürkner P.C. (2017). brms: An R Package for Bayesian Multilevel Models Using Stan. J. Stat. Softw..

[74] Bates D., Maechler M., Bolker B., Walker S. (2015). Fitting Linear Mixed-Effects Models Using lme4. J. Stat. Software.

[75] Venables W.N., Ripley B.D. (2002).

