# Supplementary material for: Uncoupling interferons and the interferon signature explains clinical and transcriptional subsets in SLE
Source: Cell Rep Med. 2024 May 13;5(5):101569. doi: 10.1016/j.xcrm.2024.101569 (PMC11148857; doi:10.1016/j.xcrm.2024.101569)
Supplement: Document S1. Figures S1 and Tables S1‒S6 [file mmc1.pdf]

Cell Reports Medicine, Volume *5*

Supplemental information

Uncoupling interferons and the interferon signature explains clinical and transcriptional subsets in SLE

Eduardo Gómez-Bañuelos, Daniel W. Goldman, Victoria Andrade, Erika Darrah, Michelle Petri, and Felipe Andrade

Supplemental Information for

Uncoupling interferons and the interferon signature explain clinical and transcriptional subsets in SLE

Eduardo Gómez-Bañuelos*, Daniel W. Goldman, Victoria Andrade, Erika Darrah, Michelle Petri, Felipe Andrade*

*Corresponding author. Email: jgomezb1@jhmi.edu and andrade@jhmi.edu

Contents

Figure S1. Correlation between activity levels of IFN-I, IFN-II and IFN-III in serum and plasma using HEK-Blue^{TM} cells. Related to Figure 1.

Table S1. Demographic characteristics of SLE patients from SPARE. Related to Figure 1.

Table S2. Association between IFN-I activity, disease duration and disease activity. Related to Figure 2.

Table S3. Association between IFN-II activity, disease duration and disease activity. Related to Figure 2.

Table S4. Association between IFN-III activity, disease duration and disease activity. Related to Figure 2.

Table S5. Associations between clinical traits captured at time of visit and activity levels of IFN types. Related to Figure 3.

Table S6. Clinical associations between elevated IFN type groups and SLEDAI activity items. Related to Figure 3.

Supplemental Excel Table 1. Differentially expressed transcripts between individual and combined IFN types in SLE. Related to Figure 4.

Supplemental Excel Table 2. Weighted correlation network analysis (WGCNA) defined modules. Related to Figure 4.

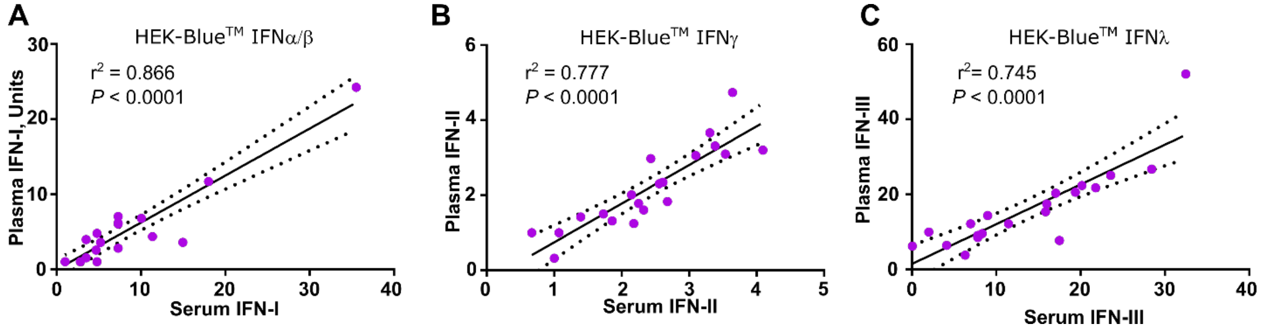

Figure S1. Correlation between activity levels of IFN-I, IFN-II and IFN-III in serum and plasma using HEK-Blue™ cells. Related to Figure 1. (**A-C**) HEK-Blue™ IFN-α/β (**A**), HEK-Blue™ IFN-γ (**B**) and HEK-Blue™ IFN-λ (**C**) were incubated with serum or plasma collected in parallel from 20 consecutive patients with SLE. r^2 was calculated using a linear regression model with plasma IFN activity as dependent variable.

Table S1. Demographic characteristics of SLE patients from SPARE. Related to Figure 1.

**Variable**	**n**	**n (%)**
Female Sex	190	177 (93%)
Race	190	
White		100 (53%)
Black		74 (39%)
Asian		9 (4.7%)
Other		7 (3.7%)
Smoking	190	15 (7.9%)
SLEDAI	187	2 (0, 15)[1]
Renal SLE	190	99 (52%)
Sjogren's Syndrome	190	48 (25%)
Anti-DNA	190	123 (65%)
Anti-Sm	189	38 (20%)
Anti-Ro52	190	75 (39%)
Anti-La	189	28 (15%)
Anti-RNP	189	52 (28%)
Anti-Ro52$_{EX4}$	190	95 (50%)
Anti-Ro52γ	190	41 (22%)
Anti-DNase1L3	156	47 (30%)
Current treatment		
Prednisone	190	68 (36%)
Hydroxicholoroquine	190	167 (88%)
Cytotoxic treatment	190	117 (62%)

[1]Median (min-max). Cytotoxic treatment includes Cyclophosphamide, Mycophenolic acid, Azathioprine, and Methotrexate.

Table S2. Association between IFN-I activity, disease duration and disease activity. Related to Figure 2.

	IFN-I, AU/mL		
	Model 1<br>β (95% CI)	Model 2<br>β (95% CI)	Model 3<br>β (95% CI)
Disease duration, yrs	-0.004 (-0.007, -0.001)		-0.004 (-0.007, -0.001)
	p = 0.020		p = 0.021
SLEDAI		0.027 (0.016, 0.037)	0.026 (0.016, 0.036)
		p <0.0001	p <0.0001
Constant	0.630 (0.555, 0.705)	0.485 (0.440, 0.531)	0.557 (0.481, 0.632)
	p <0.0001	p <0.0001	p <0.0001
Observations	322	322	322

Associations were determined using a mixed-effects linear model.

Table S3. Association between IFN-II activity, disease duration and disease activity. Related to Figure 2.

	IFN-II, AU/mL		
	Model 1<br>β (95% CI)	Model 2<br>β (95% CI)	Model 3<br>β (95% CI)
Disease duration, yrs	0.003 (-0.00001, 0.005)		0.003 (0.0001, 0.005)
	p = 0.051		p = 0.041
SLEDAI		0.006 (-0.003, 0.015)	0.007 (-0.002, 0.016)
		p = 0.172	p = 0.134
Constant	0.643 (0.583, 0.703)	0.678 (0.639, 0.717)	0.623 (0.558, 0.688)
	p <0.0001	p <0.0001	p <0.0001
Observations	322	322	322

Associations were determined using a mixed-effects linear model.

Table S4. Association between IFN-III activity, disease duration and disease activity. Related to Figure 2.

	IFN-III, AU/mL		
	Model 1<br>β (95% CI)	Model 2<br>β (95% CI)	Model 3<br>β (95% CI)
Disease duration, yrs	0.003 (-0.002, 0.008)		0.004 (-0.002, 0.009)
	p = 0.246		p = 0.169
SLEDAI		0.027 (0.008, 0.046)	0.028 (0.009, 0.047)
		p = 0.005	p = 0.004
Constant	0.698 (0.581, 0.816)	0.688 (0.609, 0.767)	0.616 (0.488, 0.745)
	p <0.0001	p <0.0001	p <0.0001
Observations	322	322	322

Associations were determined using a mixed-effects linear model.

Table S5. Associations between clinical traits captured at time of visit and increased activity levels of IFN types. Related to Figure 3.

	IFN types (dependent variables)					
	IFN-I		IFN-II		IFN-III	
Variable (Independent)	β	***p value***	β	***p value***	β	***p value***
Systolic BP	-0.007(-0.033,0.018)	0.567	0.004(-0.019,0.026)	0.753	-0.014(-0.195,0.167)	0.881
Diastolic BP	0.006(-0.037,0.049)	0.779	0.038(0.001,0.075)	**0.044**	-0.015(-0.318,0.285)	0.920
Race	1.046(-1.715,3.803)	0.677	1.452(-0.81,3.712)	0.281	4.58(-12.314,21.421)	0.660
Sex	0.094(-2.184,2.376)	0.935	-0.025(-1.948,1.901)	0.980	-0.119(-15.373,15.147)	0.988
LAI score	0.914(0.312,1.521)	**0.003**	0.071(-0.459,0.601)	0.791	5.058(0.714,9.401)	**0.023**
A: Fatigue	-0.047(-0.093,-0.001)	NA	0.032(-0.006,0.07)	NA	0.008(-0.278,0.294)	NA
B: Rash	1.429(0.505,2.358)	**0.003**	-0.48(-1.295,0.335)	0.247	-2.039(-8.79,4.698)	0.552
C: Joints	-0.688(-1.701,0.323)	0.182	0.307(-0.602,1.214)	0.507	2.698(-4.89,10.286)	0.485
D: Serositis	-0.3(-3.048,2.422)	0.829	-1.077(-3.569,1.409)	0.395	-1.162(-22.776,20.423)	0.916
A: Neurological	-0.629(-3.271,2.013)	0.639	-0.395(-2.629,1.839)	0.728	4.451(-13.312,22.221)	0.622
B: Renal	0.795(0.025,1.561)	**0.043**	0.614(-0.046,1.278)	0.068	7.177(2.065,12.301)	**0.006**
C: Pulmonary	NA	NA	NA	NA	NA	NA
D: Hematological	1.716(-0.016,3.449)	0.052	-1.202(-2.704,0.304)	0.117	-1.166(-13.424,11.096)	0.852
SLEDAI	0.295(0.154,0.437)	**< 0.001**	0.057(-0.066,0.18)	0.363	0.837(-0.188,1.86)	0.109
*Neurological**						
Organic brain syndrome	-0.674(-6.321,4.977)	0.815	-0.343(-5.119,4.436)	0.888	0.022(-37.992,38.044)	0.999
Cranial nerve disorder	0.207(-7.764,8.179)	0.959	0.012(-6.728,6.755)	0.997	29.593(-23.966,83.161)	0.278
Vasculitis	-0.719(-3.079,1.64)	0.549	0.124(-2.006,2.267)	0.990	-16.275(-34.258,1.682)	0.076
Renal						
Urinary casts	NA	NA	NA	NA	NA	NA
Hematuria	0.895(-1.376,3.168)	0.439	1.469(-0.546,3.481)	0.152	-3.018(-19.642,13.613)	0.721
Proteinuria	1.826(0.254,3.408)	**0.023**	0.725(-0.69,2.144)	0.314	18.118(6.281,29.958)	**0.003**
Pyuria	-0.546(-2.699,1.616)	0.619	0.952(-0.969,2.875)	0.331	-0.368(-16.601,15.864)	0.965
Arthritis	0.584(-1.009,2.183)	0.472	1.801(0.385,3.217)	**0.013**	3.051(-8.932,15.034)	0.617
Myositis	NA	NA	NA	NA	NA	NA
Immunological						
Low Complement	3.069(1.947,4.189)	**< 0.001**	-0.489(-1.502,0.521)	0.341	10.003(2.028,17.953)	**0.014**
Increased DNA binding	2.982(1.917,4.043)	**0.001**	-0.656(-1.605,0.291)	0.174	5.9(-1.361,13.196)	0.111
Cutaneous						
Rash	2.836(1.138,4.534)	**0.001**	0.006(-1.502,1.517)	0.994	2.121(-10.02,14.274)	0.731
Alopecia	0.709(-0.367,1.783)	0.196	0.088(-0.843,1.019)	0.853	1.848(-5.707,9.347)	0.630
Mucosal ulcers	-3.415(-5.94,-0.875)	**0.009**	-1.972(-4.226,0.287)	0.087	-9.478(-28.385,9.431)	0.325
Serositis						
Pleurisy	-0.723(-4.084,2.611)	0.671	-1.346(-4.388,1.694)	0.384	-5.799(-32.118,20.501)	0.665
Pericarditis	NA	NA	NA	NA	NA	NA
Hematological						
Thrombocytopenia	0.537(-4.085,5.162)	0.819	-2.424(-6.322,1.475)	0.222	-5.797(-36.888,25.303)	0.714
Leukopenia	2.97(-0.089,6.029)	0.057	-1.76(-4.446,0.927)	0.198	2.973(-19.033,24.98)	0.791
Infections						
Viral infection	-1.475(-3.595,0.644)	0.179	0.765(-1.145,2.669)	0.431	-5.038(-21.183,11.102)	0.540
Bacterial infection	-0.347(-1.814,1.123)	0.643	1.371(0.072,2.67)	**0.039**	4.773(-6.131,15.676)	0.390
Thrush	1.275(-1.897,4.519)	0.433	1.517(-1.267,4.293)	0.284	54.195(31.002,77.448)	**< 0.001**
Treatment						
Cytotoxic	0.528(-0.514,1.557)	0.318	-0.302(-1.164,0.558)	0.489	6.144(-0.386,12.626)	0.065
Hydroxychloroquine	0.127(-1.106,1.36)	0.839	-0.545(-1.586,0.497)	0.304	-6.154(-14.406,1.99)	0.139
NSAID use	-1.222(-2.426,-0.011)	**0.048**	-0.249(-1.268,0.774)	0.631	0.376(-7.411,8.24)	0.925
Clopidogrel	0.082(-2.141,2.312)	0.942	0.034(-1.849,1.922)	0.972	-6.901(-21.626,7.841)	0.358
Antihypertensive drugs	-0.421(-1.378,0.535)	0.387	0.476(-0.351,1.304)	0.258	1.955(-4.667,8.587)	0.562
Diuretic	-1.171(-2.212,-0.129)	**0.028**	0.005(-0.885,0.894)	0.991	-2.61(-9.458,4.251)	0.454
Calcium antagonists	0.141(-1.152,1.43)	0.830	0.015(-1.074,1.104)	0.978	7.232(-1.093,15.594)	0.088
Statin	-0.195(-1.266,0.877)	0.721	0.234(-0.676,1.141)	0.613	-1.923(-8.898,5.046)	0.587

β and p values were calculated using a mixed-effects linear model to control the effect of patients with repeated samples and disease duration. BP: blood pressure. LAI: Lupus activity index. *Other neurological items evaluated in the SELENA-SLEDAI such as seizures, psychosis, visual disturbance, lupus headache, and cerebrovascular accidents where not present in any patient at time of visit. Cytotoxic drugs were defined as: Leflunomide, Mycophenolate, Chlorambucil, Cyclosporin, Cytoxan, Tacrolimus, Azathioprine, Methotrexate, Rituximab, Etanercept, Abatacept or Adalimumab.

Table S6. Clinical associations between elevated IFN type groups and SLEDAI activity items. Related to Figure 3.

SLEDAI item	IFN subset	OR, 95% CI	P value
Arthritis	Normal	0.13(0.06,0.29)	<0.0001
Arthritis	I	2.13(0.18,24.93)	0.5467
Arthritis	II	2.31(0.4,13.24)	0.3481
Arthritis	II+III	2.6(0.51,13.17)	0.2495
Arthritis	I+II+III	6.35(1.24,32.34)	0.0262
Rash	Normal	0.08(0.03,0.23)	<0.0001
Rash	I	21.83(2.24,212.71)	0.0079
Rash	II	4.4(0.47,40.94)	0.1927
Rash	II+III	2.66(0.29,24.7)	0.3884
Rash	I+II+III	6.77(0.73,63.01)	0.0927
Alopecia	Normal	0.46(0.32,0.65)	<0.0001
Alopecia	I	3.61(1.26,10.41)	0.0172
Alopecia	II	1.11(0.48,2.6)	0.8028
Alopecia	II+III	1.29(0.6,2.77)	0.5089
Alopecia	I+II+III	1.62(0.68,3.83)	0.2753
Mucosal ulcers	Normal	0.21(0.1,0.45)	<0.0001
Mucosal ulcers	I	0(0,0)	<0.0001
Mucosal ulcers	II	1.2(0.23,6.23)	0.8283
Mucosal ulcers	II+III	0.26(0.03,2.62)	0.2536
Mucosal ulcers	I+II+III	0(0,0)	<0.0001
Hematuria	Normal	0.24(0.11,0.54)	<0.0001
Hematuria	I	1.8(0.15,21.1)	0.64
Hematuria	II	1.38(0.19,10.21)	0.7539
Hematuria	II+III	1.56(0.25,9.85)	0.6387
Hematuria	I+II+III	1.61(0.22,11.92)	0.6415
Proteinuria	Normal	0.23(0.11,0.48)	<0.0001
Proteinuria	I	1.81(0.15,21.16)	0.6366
Proteinuria	II	0.66(0.06,7.5)	0.7377
Proteinuria	II+III	4.26(0.86,21.17)	0.0766
Proteinuria	I+II+III	5.37(1.03,28)	0.0462
Pyuria	Normal	0.2(0.09,0.46)	<0.0001
Pyuria	I	0(0,0)	<0.0001
Pyuria	II	0.63(0.06,7.21)	0.7133
Pyuria	II+III	1.86(0.32,10.75)	0.4865
Pyuria	I+II+III	2.46(0.4,15.36)	0.3343
Low Complement	Normal	0.45(0.3,0.67)	<0.0001
Low Complement	I	2.34(0.68,8.05)	0.1757
Low Complement	II	0.68(0.21,2.15)	0.5084
Low Complement	II+III	1.11(0.43,2.87)	0.8325
Low Complement	I+II+III	6.98(2.82,17.26)	<0.0001
DNA binding	Normal	0.65(0.46,0.92)	0.0135

SLEDAI item	IFN subset	OR, 95% CI	P value
DNA binding	I	11.74(3.65,37.78)	<0.0001
DNA binding	II	0.83(0.34,2.02)	0.6769
DNA binding	II+III	0.9(0.4,2.03)	0.8051
DNA binding	I+II+III	5.46(2.41,12.38)	<0.0001
Thrombocytopenia	Normal	0.09(0.02,0.39)	0.0012
Thrombocytopenia	I	4.25(0.24,73.98)	0.3208
Thrombocytopenia	II	0(0,0)	<0.0001
Thrombocytopenia	II+III	0.69(0.04,11.91)	0.7975
Thrombocytopenia	I+II+III	0(0,0)	<0.0001
Leukopenia	Normal	0.13(0.05,0.31)	<0.0001
Leukopenia	I	0(0,0)	<0.0001
Leukopenia	II	0(0,0)	<0.0001
Leukopenia	II+III	0(0,0)	<0.0001
Leukopenia	I+II+III	1.03(0.16,6.51)	0.9714
Pleurisy	Normal	0.12(0.03,0.41)	<0.0001
Pleurisy	I	0(0,0)	<0.0001
Pleurisy	II	1.2(0.07,19.9)	0.897
Pleurisy	II+III	0.81(0.05,13.7)	0.8831
Pleurisy	I+II+III	0(0,0)	<0.0001
Vasculitis	Normal	0.11(0.04,0.34)	<0.0001
Vasculitis	I	3.92(0.23,65.77)	0.3425
Vasculitis	II	4.97(0.54,45.91)	0.1577
Vasculitis	II+III	3.24(0.35,30.15)	0.3024
Vasculitis	I+II+III	0(0,0)	<0.0001

Clinical associations between individual SLEDAI items and IFN subsets were determined by a multinomial mixed-effects logistic regression model.
